# Bottlebrush Polymer Templates for the Synthesis of Gold Nanostructures and their Applications as Photothermal Agents and SERS Substrates

**DOI:** 10.1002/smtd.202501059

**Published:** 2025-10-05

**Authors:** Deepak S. Chauhan, Hu Zhang, Jordan Robert, Malama Chisanga, Jean Michel Rabanel, Dikran Mekhjian, Charlotte Zaouter, Quoc Thang Phan, Wojciech Raj, Sergiy Patskovsky, Shunmoogum A. Patten, Éric Samarut, Jean‐François Masson, Xavier Banquy

**Affiliations:** ^1^ Faculty of Pharmacy Université de Montréal Montréal Québec H3T 1J4 Canada; ^2^ Department of Microbiology and Immunology Dalhousie University Halifax NS B3H 4R2 Canada; ^3^ Department of Pediatrics IWK Research Center Halifax NS B3H 4R2 Canada; ^4^ Department of Chemistry Institut Courtois Québec Centre for Advanced Materials (QCAM) Regroupement Québécois sur les Matériaux de Pointe (RQMP) and Centre Inter disciplinaire de Recherche sur le Cerveau et l'Apprentissage (CIRCA) Université de Montréal Montréal Québec H3C 3J7 Canada; ^5^ INRS Centre Armand‐Frappier Santé Biotechnologie Laval Québec H7V 1B7 Canada; ^6^ Department of Engineering Physics Polytechnique Montréal Montréal Québec H3T 1J4 Canada; ^7^ Centre de Recherche du Centre Hospitalier de l'Université de Montréal Department of Neuroscience Faculty of Medecine Université de Montréal Montréal Québec H3T 1J4 Canada; ^8^ Institute of Biomedical Engineering Faculty of Medicine Université de Montréal Montréal Québec H3T 1J4 Canada; ^9^ Department of Chemistry Faculty of Arts and Science Université de Montréal Montréal Québec H3T 1J4 Canada

**Keywords:** bottlebrush polymers, gold nanostructures, NIR‐II window, photothermal therapy, SERS substrates, soft‐template synthesis

## Abstract

An innovative approach is presented for the synthesis of gold photothermal agents tailored for Near Infrared light NIR‐I and NIR‐II photothermal applications using bottlebrush polymers (BB) as soft templates (BB@Au). Upon exposure to NIR‐I, (*λ*_ex_ = 808 nm) and NIR‐II (*λ*_ex_ = 1064 nm) light, the photothermal agents (BB@Au) exhibit robust photothermal effects, achieving temperatures up to 58.3 °C under 500 mW cm^−2^ NIR‐II laser irradiation. This remarkable thermal response enables efficient eradication of cancer cells in both 2D and 3D settings. Furthermore, comprehensive studies demonstrate the biocompatibility of BB@Au, as evidenced by concentration‐dependent and time‐dependent analyses. Studies conducted with zebrafish larvae further confirm their safety, showing no abnormalities in hatching, survival, and histology sections. Aside from their enhanced photothermal effects, the BB@Au significantly enhances the Raman signal of adsorbed analytes. This allows their quantification and broadens the potential applications of the BB@Au particles as substrates for small molecules biosensing. The bottlebrush‐based approach to produce novel gold nanostructures with augmented photothermal capabilities introduces a versatile strategy for developing precise and effective photothermal agents in a one pot process.

## Graphical abstract

A soft‐template approach using bottlebrush polymers (BB) enables the scalable synthesis of gold nanostructures (BB@Au) with strong near infrared II (NIR‐II) photothermal and Surface Enhanced Raman Spectroscopy  activity. BB@Au nanoparticles exhibit efficient tumor ablation in 2D and 3D models, high biocompatibility in zebrafish assays, and excellent Raman sensitivity, highlighting their promise as versatile nanotheranostic platforms for cancer treatment and sensing.

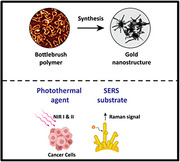

## 1 Introduction

Photothermal cancer therapy using near‐infrared (NIR) light‐activated nanoparticles has garnered considerable attention as a minimally invasive treatment method.^[^ ^1^, ^2^, ^3^, ^4^ ^]^ Gold‐based nanostructures such as nanorods,^[^ ^5^ ^]^ nanoshells,^[^ ^2^, ^3^, ^6^ ^]^ and nanostars^[^ ^7^ ^]^ have been extensively studied as photothermal agents due to their strong localized surface plasmon resonance absorption within the NIR window. Among these, gold nanoshells have advanced to Phase 3 human clinical trials for photothermal cancer therapy.^[^ ^8^, ^9^ ^]^ However, the progress of these and other nanoparticles‐based photothermal agents has been remarkably slow due to several challenges, such as the absence of standardized large‐scale synthetic methods to produce photothermal agents with consistent properties, concerns about potential toxicity, and the specific limitations associated with near‐infrared window‐I (NIR‐I) based photothermal agents. The absence of standardized large‐scale synthetic methods has resulted in considerable variability in the physicochemical properties of synthesized nanoparticles. This variability, encompassing size, shape, and absorbance wavelengths, not only constitutes a formidable challenge to reproducibility but also complicates the translational potential of these agents. Achieving a consistent and scalable synthetic pathway is crucial for advancing these agents from experimental studies to clinical applications.^[^ ^10^ ^]^

Moreover, concerns regarding the potential toxicity of capping agents and surfactants used in synthesis protocols have cast doubts on the safety profile of nanoparticle‐based photothermal agents. This issue adds layer of complexity to the regulatory approval process, necessitating thorough evaluation and slowing down the translation of these agents to clinical use.^[^ ^11^ ^]^ Furthermore, the inherent limitations associated with NIR‐I based photothermal agents have become apparent with more exhaustive investigation: while effective for certain applications, NIR‐I has its constraints, including limited tissue penetration and potential interference from background signals.^[^ ^12^, ^13^ ^]^ To address these limitations, there is a growing demand for the development of near‐infrared window‐II (NIR‐II) based photothermal agents, which offer enhanced tissue penetration capabilities and reduced background signal interference.^[^ ^13^ ^]^ The transition to NIR‐II based agents necessitates a reevaluation of synthetic methods and an exploration of novel materials that can harness the benefits of this extended wavelength range.

To address these challenges, the use of soft templates, such as bovine serum albumin (BSA),^[^ ^14^, ^15^ ^]^ deoxyribonucleic, DNA^[^ ^16^ ^]^ and peptides,^[^ ^17^ ^]^ specifically bottlebrush (BB) polymers,^[^ ^18^ ^]^ has emerged as a promising strategy in the synthesis of inorganic nanostructures. BB polymers are a class of macromolecules characterized by a central polymer backbone densely grafted with side chains.^[^ ^19^ ^]^ Their unique architecture allows them to serve as versatile soft templates, offering precise control over the shape, size, and surface characteristics of nanomaterials.^[^ ^20^ ^]^ In recent years, BB polymers have been utilized as soft templates for the controlled synthesis of gold nanoparticles, demonstrating their ability to stabilize and guide the formation of nanoparticles with catalytic properties.^[^ ^21^ ^]^ The densely packed side chains of BB polymers provide steric stabilization, preventing aggregation, which is essential for reproducible, uniform, and monodisperse synthesis.^[^ ^22^ ^]^ Additionally, the chemical versatility of BB polymers allows for the functionalization of their side chains with specific ligands, enhancing the biocompatibility as well as targeting capabilities of the synthesized nanoparticles.^[^ ^23^, ^24^ ^]^

Herein, a novel library of photothermal agents called BB@Au(s), produced using BB polymers as soft templates, was designed to operate in the NIR‐I (*l*_ex_ = 808 nm) and NIR‐II (*l*_ex_ = 1064 nm) windows, offering enhanced photothermal therapy and promising potential for photo‐immunotherapy and Surface Enhanced Raman spectroscopy (SERS) applications. BB polymers were strategically chosen due to their unique architectural features, dense, radially oriented side chains, and tunable backbone lengths, which provide well‐defined nanoreactors for controlling the nucleation and growth of gold nanostructures.^[^ ^25^ ^]^ This morphogenic behavior enables precise tuning of size, shape, and plasmonic properties, especially in the biologically relevant NIR‐I and NIR‐II regions, which is often difficult to achieve using traditional linear polymers or surfactants. While soft template approaches using dendrimers, block copolymers, and micelles have been explored for nanoparticle synthesis, the use of BB polymers in this context remains largely unexplored.^[^ ^26^ ^]^ To the best of our knowledge, this is the first report leveraging BB polymers to direct the synthesis of NIR‐active gold nanostructures for photothermal and Surface Enhanced Raman Spectroscopy (SERS) applications. The preliminary analyses demonstrate that these photothermal agents exhibit comparable photothermal conversion and biocompatibility to the previously reported photothermal agents.^[^ ^14^, ^15^ ^]^ Additionally, we show that these gold nanostructures can also be used as substrates for the quantification of low concentrations of analytes by SERS. The end results of this research work aim to overcome current translational roadblocks by establishing an easy, tunable, scalable, and non‐toxic synthesis pathway for producing promising photothermal agents relevant for clinical translation and multimodal biomedical applications.

## 2 Results and Discussion

### 2.1 Synthesis and Characterization of BB@Au(s)

To synthesize well‐controlled BB polymers as a template, we employed a “grafting‐from” approach utilizing as macroinitiators a set of block copolymers incorporating methyl methacrylate (MMA) and 2‐hydroxyethyl methacrylate (HEMA) with a trimethylsilyl (TMS) protection group (referred to as P(MMA‐*co*‐HEMA‐TMS)) synthesized via atom transfer radical polymerization (ATRP).^[^ ^27^, ^28^, ^29^, ^30^, ^31^ ^]^ The control of the number of repeating units in the macroinitiator was achieved by adjusting the feed ratio of methyl methacrylate (MMA) and 2‐(trimethylsilyloxy)ethyl methacrylate (HEMA‐TMS) to the bromide initiator, in conjunction with the polymerization reaction time. Throughout the synthesis, the in‐feed ratio of the two monomers, MMA and HEMA‐TMS, was consistently maintained at 1:1. Optimal conditions, involving the mole ratio of monomer to initiator and conversion ratio, were selected to ensure well‐controlled polymerization with minimal polydispersity. Three copolymers with different molecular weights were synthesized (Table S1, Supporting Information) following this synthesis procedure (scheme S1, Supporting Information). For the polymer P(MMA‐*co*‐HEMA‐TMS)_418_, the reaction was halted at a conversion ratio of 19.5%, yielding a final polymer comprising 418 repeat units. By increasing the monomers/initiator in‐feed ratio to 2000 while maintaining the same conversion level, the final backbone repeating units for the polymer rose to 880. At a conversion ratio of 40.9%, a polymer with 1636 backbone units was obtained. A comprehensive summary of the in‐feed and conversion ratios, along with the final values for backbone repeating units, is presented in Table S1 (Supporting Information). NMR analysis of the purified polymer, depicted in Figure S1 (Supporting Information), revealed a ratio of the two monomers matching the 1:1 in‐feed ratio, confirming the precise control achieved during the polymerization process using the ATRP method.

The removal of the trimethylsilyl (TMS) groups from the three polymers was accomplished using tetra‐n‐butylammonium fluoride (TBAF). Subsequently, the deprotected hydroxyl group underwent a reaction with 2‐bromoisobutyryl bromide, resulting in the formation of the polymer macro‐initiator. A compelling correlation in the chemical shifts of peaks *e* and *d*, as observed in Figures S1 and S2 (Supporting Information), robustly supports the conversion of the TMS group into a bromide group. Utilizing these three macro‐initiators, side chain poly(2‐(dimethylamino ethyl)methacrylate) (PDMAEMA) was grafted^[^ ^32^ ^]^ (Figure S3, Supporting Information). The in‐feed ratio, conversion ratio, and final repeating units of the pendant chain are comprehensively detailed in Table S2 (Supporting Information). Consequently, three P(MMA‐*co*‐BiBEM)‐*g*‐PDMAEMA bottlebrush polymers were successfully synthesized. In a singular‐step reaction, facilitated by the transformation of the tertiary amine, the three polymers underwent quaternization, resulting in final charged polymers, P(MMA‐*co*‐BiBEM)‐*g*‐qPDMAEMA, used as a template for the growth of gold nanostructures (Table S3 and Figure S4, Supporting Information). For clarity, we have adopted abbreviated names, BB_400_, BB_800_, and BB_1600_, for convenience in referring to the three polymers, P(MMA‐*co*‐BiBEM)_418_‐*g*‐PDMAEMA_76_, P(MMA‐*co*‐BiBEM)_880_‐*g*‐PDMAEMA_60_, and P(MMA‐*co*‐BiBEM)_1636_‐*g*‐PDMAEMA_90_, respectively (Table S3, Supporting Information).

**Figure 1:**
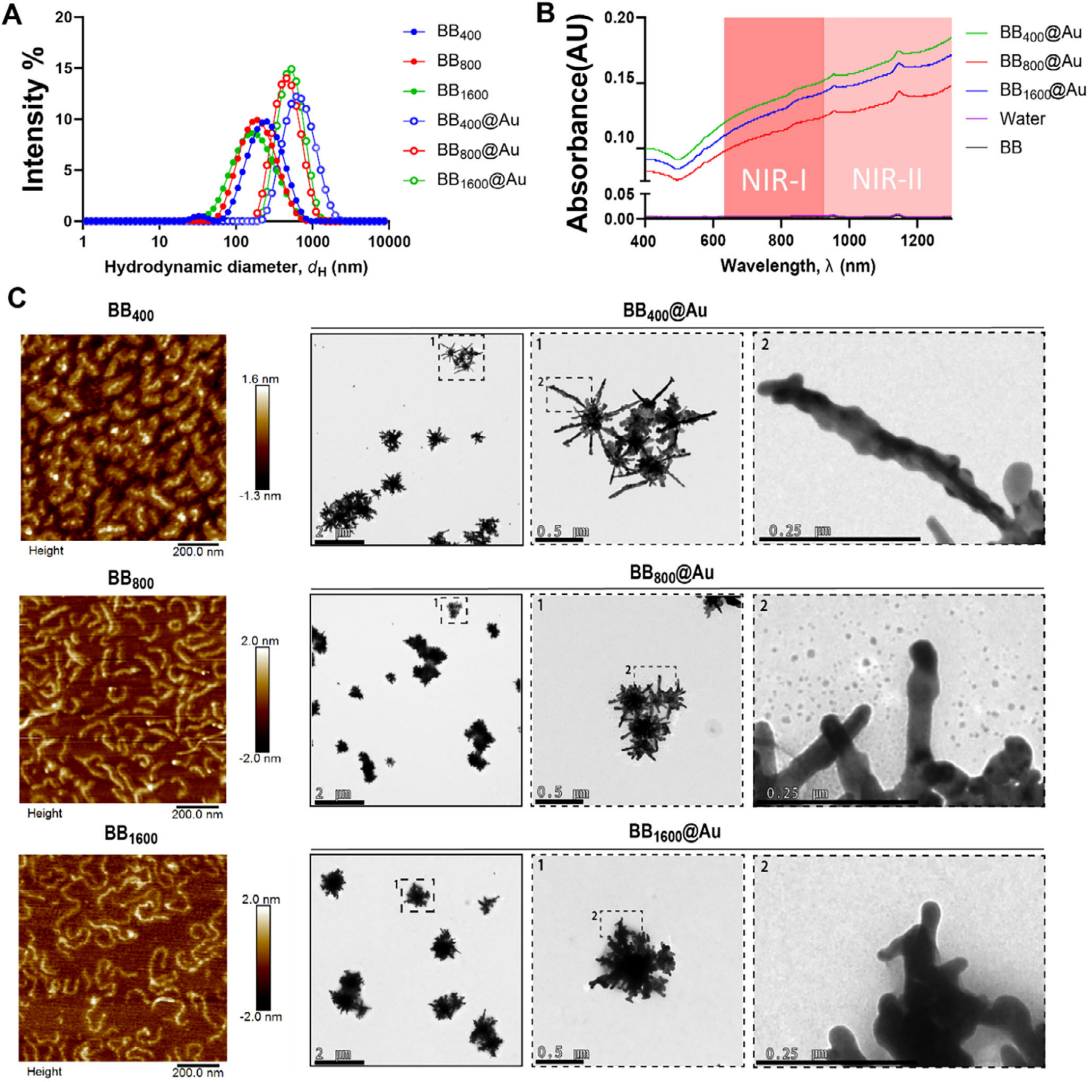
Characterization of bottlebrush polymers templates and the corresponding BB@Au nanoparticle. A) Hydrodynamic diameter of BB_400_, BB_800_, BB_1600_, BB_400_@Au NPs, BB_800_@Au NPs, and BB_1600_@Au NPs. B) Absorbance profile of BB, BB_400_@Au NPs, BB_800_@Au NPs, BB_1600_@Au NPs, and water (optical path length for all samples was 1 mm). C) AFM images of BB polymers on mica surfaces and TEM images of BB@Au NPs with corresponding magnified images shown in dashed rectangles.

**Figure 2:**
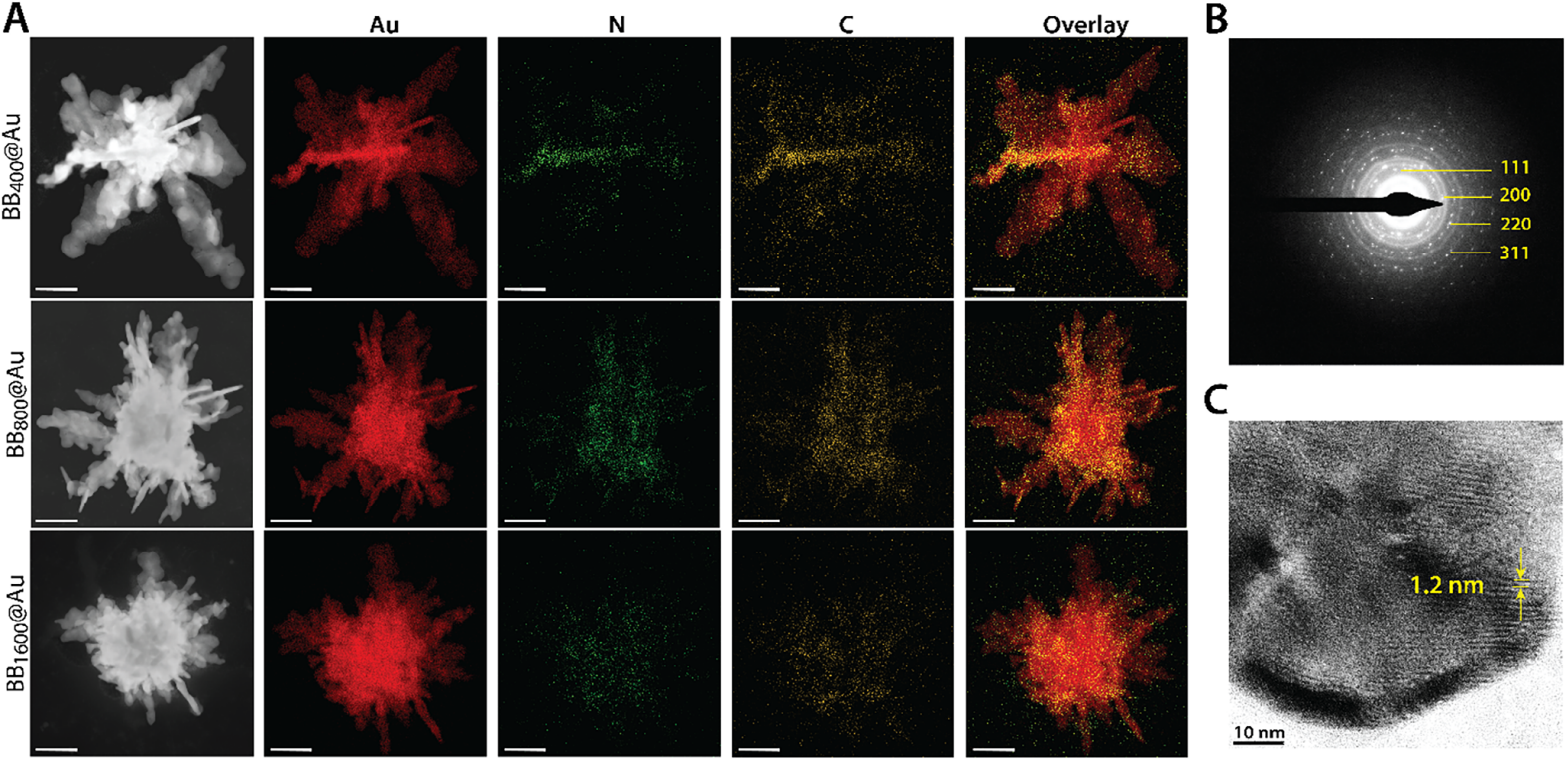
Comparative elemental mapping analysis, and diffraction pattern of BB_400_@Au, BB_800_@Au, and BB_1600_@Au. A) Elemental mapping of BB@Au_s_, the red, yellow, and green colors show the distribution of gold, nitrogen, and carbon, respectively. B) The diffraction pattern, and C) lattice pattern of a BB_400_@Au particle.

The synthesis of photothermal agents, BB@Au NPs, employed an in‐situ gold coating method,^[^ ^33^, ^34^ ^]^ a soft template‐driven approach utilizing the synthesized BB polymer as the template. The synthesis pathway is illustrated in Figure S5 (Supporting Information) and a digital image of the samples is provided in Figure S8A (Supporting Information). The resulting BB@Au NPs were characterized by different techniques to elucidate their structure, morphology, composition, and surface properties, essential for assessing their biocompatibility and photothermal behavior. The hydrodynamic sizes and (polydispersity index) in water of BB_400_, BB_800,_ and BB_1600_ were 93 nm (0.07), 93 nm (0.07), and 115 nm (0.22), respectively. The nucleation and growth of gold nanostructure was successfully verified by hydrodynamic size measurement. Upon gold deposition, the sizes of the corresponding BB_400_@Au NPs, BB_800_@Au NPs, and BB_1600_@Au NPs increased to 345 nm (0.19), 333 nm (0.20), and 353 nm (0.21), respectively, as shown in **Figure** **1**A. The absorbance spectra of the BB@Au NPs were recorded using a UV–vis–NIR spectrophotometer and compared to controls, i.e., BB polymers and water. Notably, all BB@Au NPs exhibited significant absorbance in the NIR‐I and NIR‐II regions, crucial for applications like photothermal therapy or imaging where deep tissue penetration is necessary, as shown in Figure 1B. BB_400_, BB_800_, and BB_1600_ were further characterized using atomic force microscopy (AFM) to examine their conformation and morphology (Figure 1C). As shown in the AFM images, as the BB backbone length increases, the polymer tends to coil on itself, as already reported^[^ ^35^ ^]^ and transitions from a conformation described as “rigid ellipsoid” to “flexible cylinder”. Following the nucleation and growth of gold nanostructures^[^ ^33^, ^34^ ^]^ on the BB polymer, BB_400_@Au NPs exhibited a “*nanoaster*” shape characterized by multiple thin wire‐like projections emerging from the core of the particle. BB_800_@Au NPs still presented a similar structure with shorter projections, and BB_1600_@Au NPs transformed into a rough spherical shape with fewer projections and very short, as illustrated in Figures 1C and 2 and Figures S5,S6 (Supporting Information).

The composition and structure of BB_400_@Au, BB_800_@Au_,_ and BB_1600_@Au NPs were further investigated using elemental mapping. Mapping of carbon and nitrogen elements on individual BB@Au particles revealed a strong effect of the BB length on the conformation of the BB template in the final particle. For the BB_400_@Au particles, carbon and nitrogen distribution delineate a rod‐like shape at the center of the particle (**Figure** **2**A). In comparison, carbon and nitrogen were uniformly distributed across the surface of BB_800_@Au and BB_1600_@Au NPs (Figure 2A). These observations align well with the AFM images shown in Figure 1C where long BB chains systematically appeared to coil on themselves while shorter chains always exhibit a straight conformation. These data provide detailed insights into how the conformation of the BB template dictates the final structural characteristics of the BB@Au particles.

The energy dispersive X‐ray spectroscopy (EDX) analysis and selected area electron diffraction (SAED) results of BB@Au(s) revealed notable differences in the crystallographic composition of gold on different BB polymers. The varying intensities of the Au‐M, Au‐Lα, and Au‐Lβ bands across BB_400_@Au, BB_800_@Au, and BB_1600_@Au suggest that the length of the BB polymers influences not only the structural arrangement but also the elemental distribution of gold on these nanostructures, Figure S8B (Supporting Information). For instance, BB_400_@Au, with the highest intensity of the Au–M band, indicates a more pronounced gold presence in its core structure, which could be behind its enhanced photothermal properties due to the dense packing of gold atoms.^[^ ^36^, ^37^ ^]^ In contrast, BB_800_@Au shows a lower Au‐M intensity but an increased Au‐Lβ band, indicating a shift in the gold distribution, possibly leading to a more surface‐oriented arrangement.^[^ ^37^ ^]^ This could also affect the photothermal efficacy, as surface gold atoms tend to have more significant plasmonic activity.^[^ ^38^, ^39^ ^]^ BB_1600_@Au, with intermediate intensities across all gold bands, suggests a more balanced distribution of gold across the structure, likely because the longer BB polymer allows for more uniform gold nucleation and growth. This uniformity might help in offering stable photothermal performance while reducing overheating noises, which is crucial for minimizing damage to surrounding tissues during preclinical and clinical studies. The gold distribution differences could also impact the biocompatibility and circulation time of BB@Au(s) in biological systems. Also, BB@Au(s) with more surface gold, like BB_800_@Au, might exhibit higher interaction with biological proteins, potentially leading to faster clearance from the body, whereas those with denser core gold distribution, like BB_400_@Au, may show more prolonged blood circulation due to reduced surface interaction with the immune system.^[^ ^40^, ^41^ ^]^

The presence of gold on the BB surfaces was further confirmed using diffraction patterns and high‐resolution field emission gun‐transmission electron microscope (FEG‐TEM) images, as shown in Figure 2C. Periodic fringes with a spacing of ≈1.2 nm were observed, which are attributed to Moiré patterns resulting from overlapping crystalline gold domains or the interaction between the gold lattice and the underlying BB polymer matrix, as shown in Figure 2C. These interference patterns, along with the disturbance of the [220] diffraction spots and the appearance of 12‐ or 24‐fold patterns in the selected area electron diffraction (SAED), support the hypothesis that the coalescence of gold seeds on the BB polymer surface is the primary mechanism driving the formation of BB@Au nanoparticles.

### 2.2 Stability of BB@Au(s)

**Figure 3:**
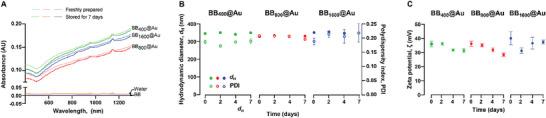
Stability of BB@Au NPs in solution. A) UV–vis–NIR Spectra of BB@Au particles in water after 0 day and 7 days of incubation at room temperature (optical path length for all samples was 1 mm); B) Hydrodynamic size of BB@Au particles measured by DLS at different incubation times over one week at room temperature; C) Zeta potential of BB@Au particles after 0 day, 2 days, 4 days, and 7 days incubation in water. Data are presented as mean ± SD (*n* = 3).

Before characterizing the photothermal properties of BB@Au NPs, their stability was assessed by examining changes in absorbance, zeta potential, and hydrodynamic diameter over time at room temperature, as shown in **Figure** **3**. BB_400_@Au, BB_800_@Au_,_ and BB_1600_@Au NPs showed no changes in absorbance from the day of synthesis (day 0) to after 7 days, as shown in Figure 3A. This consistency suggests that the optical properties of the BB@Au NPs remained stable over this period, suggesting no significant alterations in their structural integrity nor composition that might impact their absorbance characteristics. Furthermore, the hydrodynamic diameter and polydispersity of BB@Au NPs were measured at different incubation time, as shown in Figure 3B. It was noticed that there were no definitive changes in the hydrodynamic diameter or polydispersity of any BB@Au NPs. In the case of BB_400_@Au, the polydispersity index (PDI) remained nearly unchanged from its initial value of 0.19, indicating minor fluctuations in size without any significant modifications. In the analysis of zeta potential, the values for BB@Au NPs ranged between 28 and 40 mV, as depicted in Figure 3C. For BB_400_@Au, BB_800_@Au NPs, there was a slight decrease in zeta potential values over time, starting from 37 mV and decreasing to 27 mV. This trend could indicate the gradual desorption of positively charged organic material, such as the BB template. However, for BB_1600_@Au NPs, this trend was not observed consistently, as there was significant variability in the measurements, for which we currently lack explanations.

These findings collectively suggest that the BB@Au NPs maintained consistent optical properties, surface charge, and size characteristics over the evaluated time period. These stable properties are crucial for ensuring reliability and consistency in subsequent analyses, especially in applications such as photothermal therapy, where precise optical and physical properties are essential.

### 2.3 Biocompatibility of BB@Au(s)

**Figure 4:**
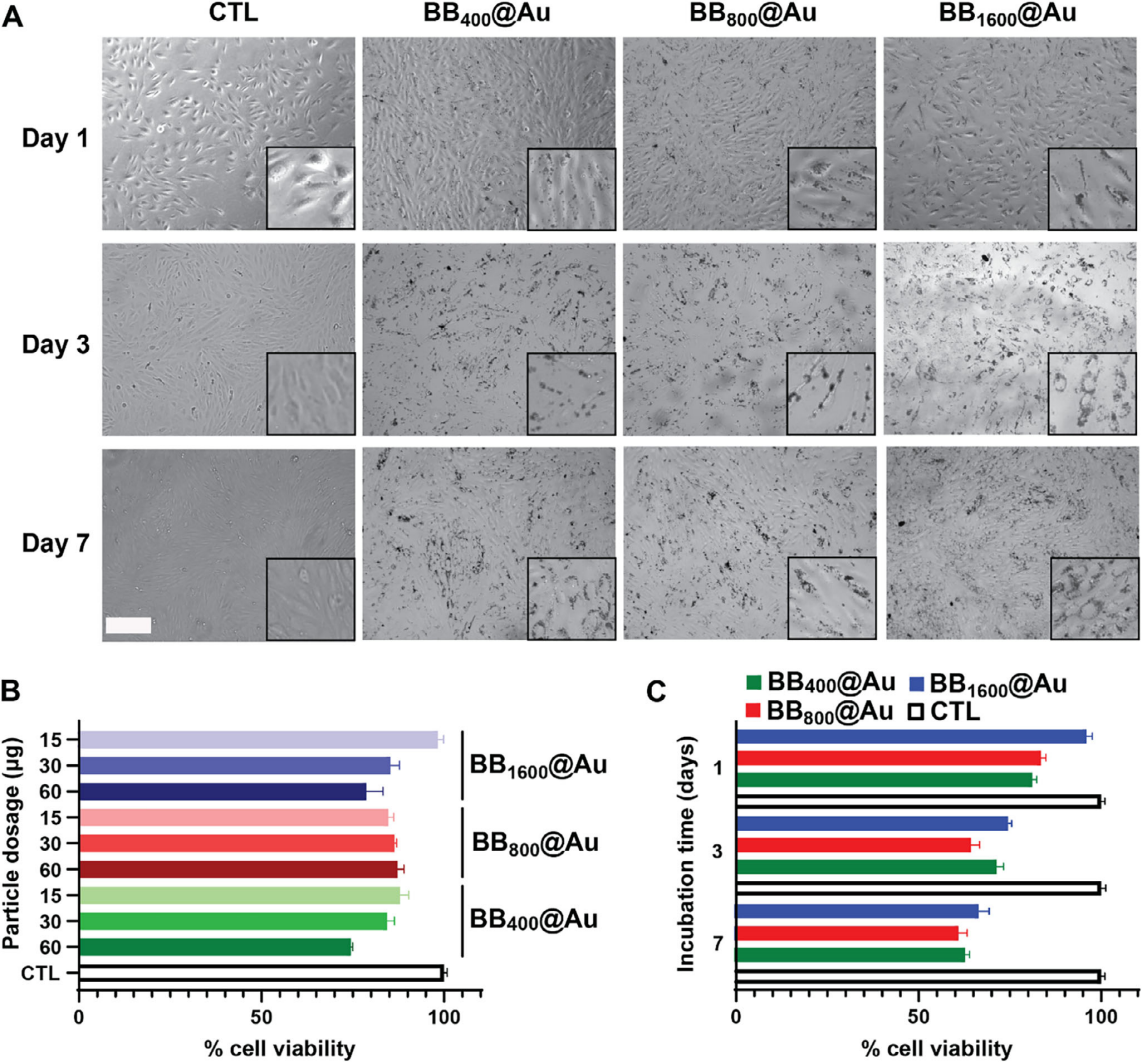
The biocompatibility assessment of BB@Au particles. A) Bright field imaging of hCMEC/D3 BBB cells at different time points after incubation with BB_400_@Au, BB_800_@Au_,_ and BB_1600_@Au at 60 µg, the insets images showing magnifications of the cells; scale bar is 100 µm. B) The percentage of cell viability in response to different concentrations and C) the number of incubation days. Data are presented as mean ± SD (*n* = 3).

Biocompatibility of the different BB@Au NPs was tested in vitro and in vivo before functional tests. In vitro, NPs were incubated with brain microvascular endothelial cells hCMEC/D3 at increasing concentrations to determine the maximum tolerated dose (**Figure** **4**A). This cell line was specifically chosen because it models the blood–brain barrier (BBB) endothelium, which is a critical interface for delivering therapeutics to brain tumors such as glioblastoma. For BB_400_@Au NPs, cell viability reached 80% at the highest tested dose (60 µg for 10^4^ cells) while it stayed above 80% for BB_800_@Au and BB_1600_@Au NPs at the same dosage (Figure 4B). Such high biocompatibility, especially at elevated concentrations, is an encouraging sign for potential therapeutic uses where controlled dosages and minimal cytotoxicity are critical factors for ensuring safe and effective treatments.

Cell viability was also evaluated at different incubation time points, at the highest dosage of 60 µg per 10^4^ cells. The results, as shown in Figure 4C, present a detailed assessment of cell viability percentages over a span of 1, 3, and 7 days for each BB@Au NPs. For all the NPs, cell viability decreased from 80% down to 60% over a period of 7 days, indicating a gradual reduction in cellular health. Cellular morphology at 7 days remained similar to that observed at 1 day and in control (untreated cells), as displayed in Figure 4A. The stability of cell morphology despite reduced proliferation over time highlights that the concentration of gold and its distribution on the BB polymers might play a role in minimizing immediate damage. Notably, the higher biocompatibility of BB_800_@Au and BB_1600_@Au suggests that larger or more uniformly coated gold nanoparticles (due to the longer BB polymers) might present more favorable biological interactions, possibly due to better dispersion of gold across the nanoparticle surface and reduced sharpness of structures, which are known to influence cell uptake and cytotoxicity.^[^ ^42^, ^43^ ^]^

**Figure 5:**
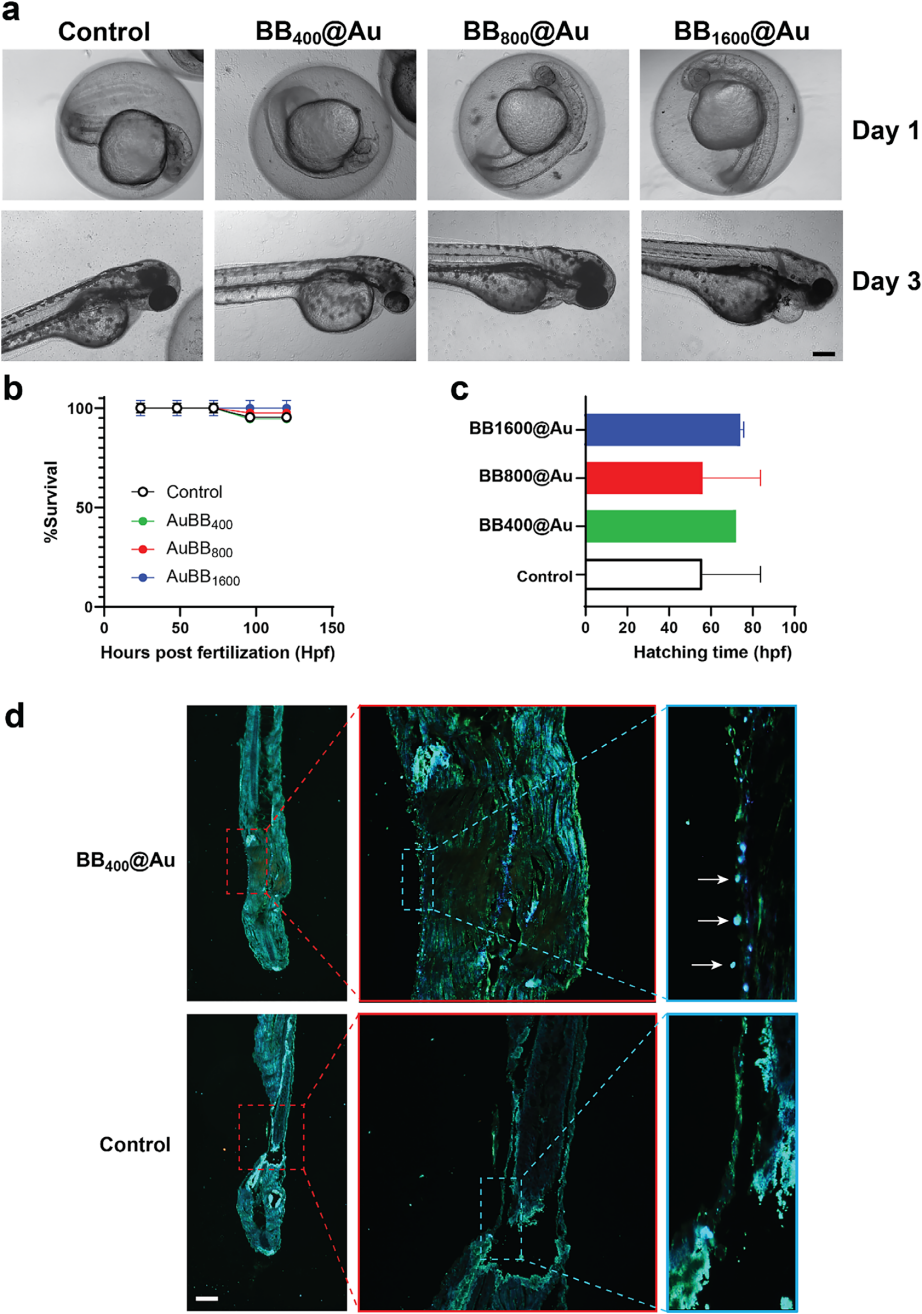
Biocompatibility assessment in zebrafish larvae. A) 5X bright field microscopy images of zebrafish eggs and larvae exposed to BB@Au particles taken at different time points; scale bar is 200 µm. B) The complete hatching time of the eggs exposed to the different formulations; C) Percentage of larvae survival without abnormality at Day 5. D) The side illumination microscopy (SIM) of the histological longitudinal section of 5‐day old zebrafish larva incubated with BB_400_@Au particles. The SIM of longitudinal section of 5‐day old zebrafish larva incubated with the BB_400_@Au, and control (i.e., only fish water instead of BB_400_@Au particles) at 40X and 60X (dashed rectangles). The BB_400_@Au particles are reflected as bright spots on the gills of the zebrafish (arrows); scale bar is 200 µm. Data are presented as mean ± SD (*n* = 3).

While in vitro toxicity analyses like these offer valuable insights into cellular responses and allow for controlled, reproducible screening, they inherently lack the complexity of living systems, including systemic interactions, metabolic processes, and immune responses, which may influence nanoparticle behavior and toxicity.^[^ ^44^ ^]^ To complement these findings and better predict biocompatibility in physiological environments, in vivo assessments were performed using zebrafish larvae (Figure S8, Supporting Information). The larvae were exposed to the NPs (60 mg per 20 larvae in 2 mL). It was observed that prolonged exposure did not induce any significant effect on the hatching time or percentage of survival of the zebrafish larvae, as illustrated in **Figure** **5**B,C. For BB_400_@Au and BB_1600_@Au_,_ 100% hatching was completed at 70 h postfertilization (hpf), while for BB_800_@Au_,_ 100% hatching occurred by 55 hpf like control.

The percentage of survival of all the treated groups was similar to the control. Moreover, microscopy images of the zebrafish larvae at different exposure times did not reveal any abnormalities statistically, as shown in Figure 5A. This observation was further confirmed by histology (Figure S9, Supporting Information). These detailed findings suggest a high degree of biocompatibility of the BB@Au NPs with zebrafish larvae under the conditions tested. This information is vital in assessing the safety profile of AuBBs, laying a foundation for further exploration and potential applications in biomedical where biocompatibility with living organisms is a crucial consideration.

The BB@Au particles uptake and localization within the zebrafish larvae were characterized by side illumination microscopy (SIM). SIM allows to localize the particles within the larva without the need of labeling them. Histology sections of zebrafish revealed that BB_400_@Au was uptaken by the zebrafish via its gills. BB_400_@Au was found lying on the surface of gills as bright spots (see dashed rectangles in Figure 5) in the 40 X and 60 X magnification in comparison to the control, as shown in Figure 5 and Figure S11 (Supporting Information). The uptake and presence of BB_400_@Au on the surface of gills suggest a preferential route of entry for these nanoparticles within the zebrafish larvae during the incubation period. The localization of these nanoparticles as bright spots on the surface of the gills suggests a size‐dependent uptake mechanism, a phenomenon reported in earlier studies.^[^ ^45^, ^46^ ^]^ The presence of BB_400_@Au on the gills, as opposed to other body parts, suggests that smaller gold nanoparticles are preferentially taken up through the respiratory route in aquatic organisms. The tail fin of the zebrafish was also characterized using SIM, and the presence of BB@Au(s), to a lesser extent, was further confirmed using scanning electron microscope (SEM) and elemental analysis, as shown in Figure S10 (Supporting Information). This secondary localization suggests that while gills serve as the primary route of nanoparticle entry, other body parts, such as fins can also interact with these nanoparticles. This distribution pattern is in line with the previous finding, wherein nanoparticle accumulation in multiple tissues, including fins and gills, depends on nanoparticle size and surface charge.^[^ ^47^, ^48^, ^49^ ^]^

### 2.4 Photothermal Properties of BB@Au(s)

**Figure 6:**
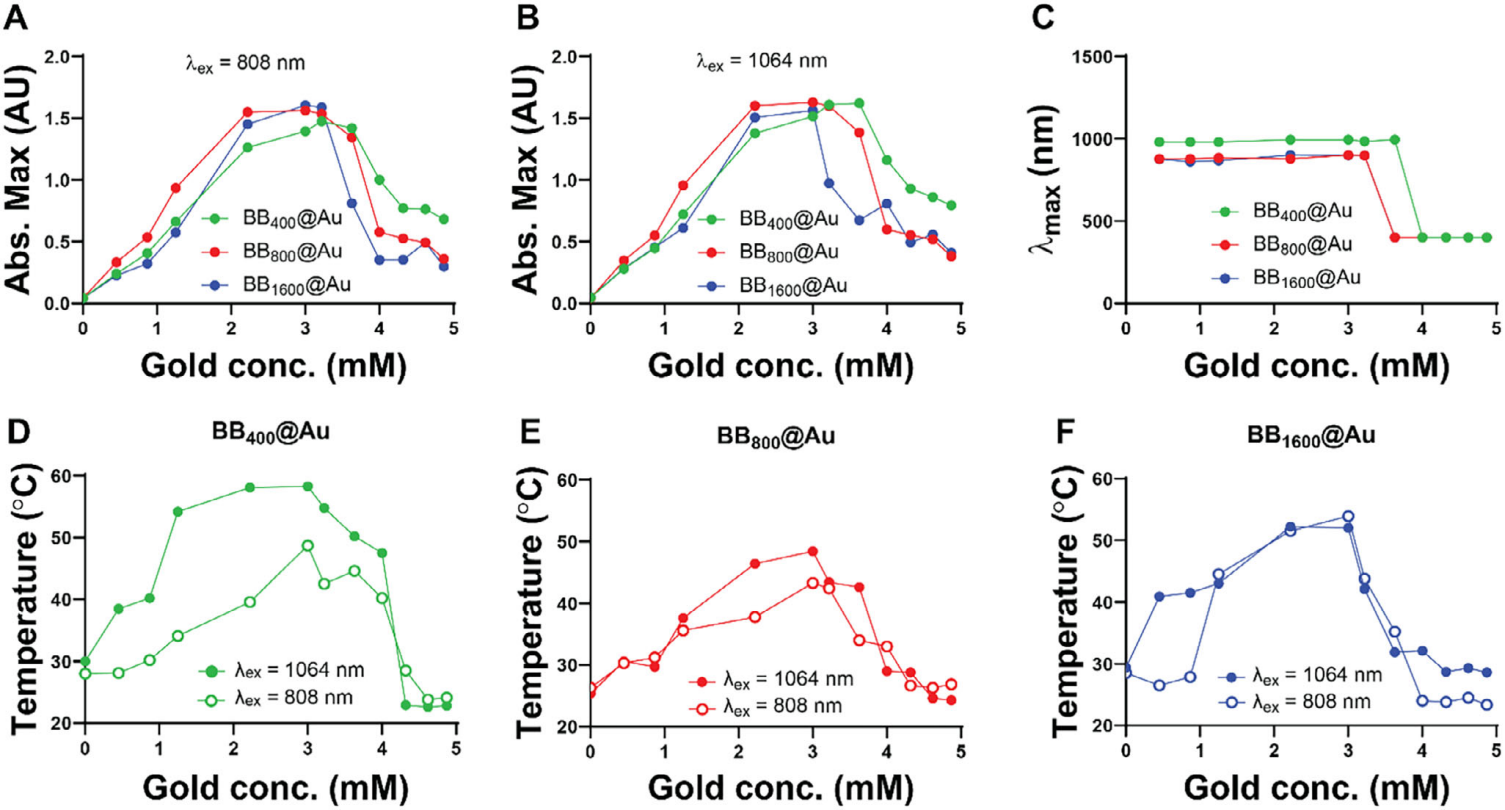
The photothermal properties of BB@Au particles. The maximum absorbance achieved at *λ*_ex_ = 808 nm, **A**) and *λ*_ex_ = 1064 nm, B) and λ_max_ C) achieved using different concentrations of gold. The increment in temperature of BB_400_@Au D), BB_800_@Au E), and BB_1600_@Au F) on the irradiation of NIR‐I and NIR‐II laser for 5 min.

The photothermal properties of BB@Au particles were then evaluated, compared, and tested in 2D and 3D cell culture settings. The absorbance in the NIR‐I (*λ*_ex_ = 808 nm) and NIR‐II (*λ*_ex_ = 1064 nm) spectral windows was examined at different gold concentrations. The maximum absorbance at *λ*_ex_ = 808 nm was observed at gold concentrations of 3.2, 3.0, and 3.0 mm for BB_400_@Au, BB_800_@Au, and BB_1600_@Au, respectively. Meanwhile, the maximum absorbance at *λ*_ex_ = 1064 nm was observed at gold concentrations of 3.6 mm for BB_400_@Au, and 3 mm for BB_800_@Au_,_ and BB_1600_@Au, as shown in **Figure** **6**A–C.

Notably, high concentrations of gold led to a reduction of the absorbance intensity across all BB@Au suspensions (Figure 6A–C), hinting at a possible saturation effect or changes in the BB@Aus' aggregation state at higher concentrations. The absorbance maximum (*λ*_max_) of AuBBs was determined by varying the concentration of gold. It was found that BB_400_@Au, BB_800_@Au_,_ and BB_1600_@Au exhibited *λ*_max_ at wavelengths of 994, 899, and 900 nm, respectively (Figure 6C).

Subsequently, the increment of temperature with respect to gold concentration and NIR‐I and NIR‐II lasers was also evaluated. It was found that on the irradiation of NIR‐I laser, the maximum temperature achieved in case of BB_400_@Au, BB_800_@Au_,_ and BB_1600_@Au was 47, 43, and 53.9 °C at the gold concentration of 3.0 mm. While on the irradiation of NIR‐II laser, the maximum temperature recorded in case of BB_400_@Au, BB_800_@Au_,_ and BB_1600_@Au, was 58.3, 48.4, and 52.2 °C at the gold conc. of 3.0, 3.0, 2.2 mm, respectively (Figure 6D–F). The notable difference in the phototransduction of NIR‐I and NIR‐II lasers for BB_400_@Au implies an enhanced surface area for laser absorption as per the structure of BB_400_@Au. Furthermore, minimal changes were observed over 3 heating and cooling cycles, suggesting excellent photostability and making these photothermal agents suitable for multiple treatment sessions (Figure S12, Supporting Information). These findings underscore the tunability of photothermal properties of BB@Au with respect to gold concentration, specific laser wavelengths, and structural differences between different types of particles. This study provides essential insights for potential applications of BB@Au in photothermal therapy, where precise and controlled heating is required, for therapeutic purposes, and paves the way for the strategic design and use of these materials in other fields.

The excellent photoconversion efficiency of BB@Au is comparable to the majority of reported photothermal agents.^[^ ^1^, ^2^, ^3^, ^5^, ^6^ ^]^ Upon photoirradiation with NIR‐I and NIR‐II lasers, the temperatures of BB_400_@Au, BB_800_@Au, and BB_1600_@Au particles increased significantly, as measured by a probe thermometer. The respective temperature elevations were 47 °C (± 2.4) and 58.3 °C (± 1.6) for BB_400_@Au, 43.3 °C (± 2.1) and 48.4 °C (± 1.8) for BB_800_@Au, and 52.2 °C (± 1.3) and 53.9 °C (± 2.5) for BB_1600_@Au. (Figure 6D–F). The photothermal efficiencies of the BB@Au were evaluated. BB_400_@Au, BB_800_@Au, and BB_1600_@Au particles exhibited a photothermal efficiency of 22.4% / 25.6%; 20.4% / 23.6%, and 23.8% / 25.1% at *λ*_ex_ = 808/1064 nm. The photothermal efficiency of all BB@Au was above 20%, which signifies that a significant proportion of absorbed light energy is effectively converted into heat.

**Figure 7:**
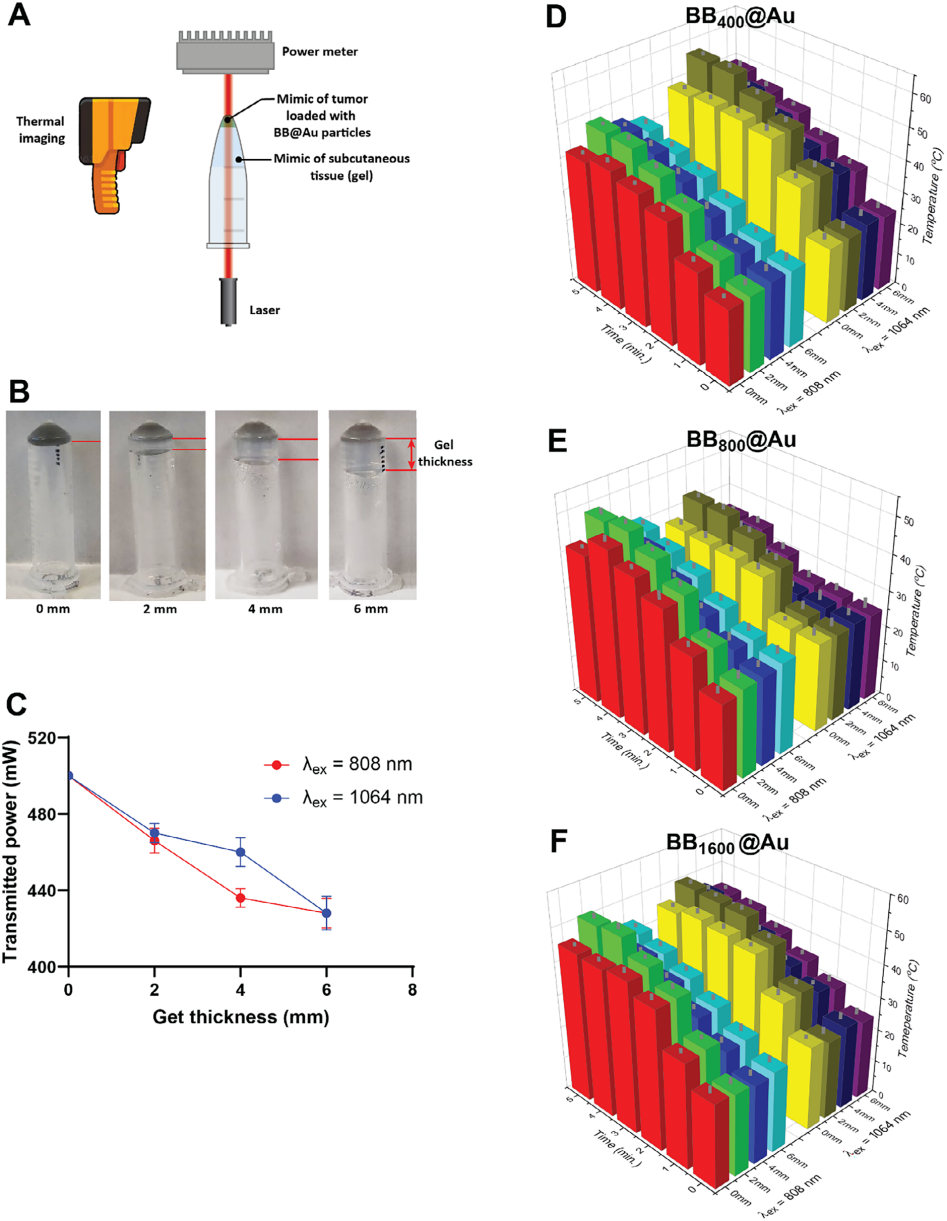
Photothermal characterization of BB@Au particles in the presence of a tissue‐mimic barrier. A) Schematic representation of the experimental setup used to quantify the photothermal activity of the BB@Au particles through tissue micmics; B) Representative digital photo showing the BB_400_@Au particles embedded in agarose gel and covered with different thicknesses of agarose gel. C) Change in laser power after passing through the agarose gel of different thicknesses. D–F) Change in temperature upon irradiation at *λ*_ex_ = 808 nm or *λ*_ex_ = 1064 nm of the different BB@Au particles embedded in agarose gels at different thicknesses of tissue mimics. Data are shown as mean ± SD (*n* = 3).

Furthermore, the photothermal properties of BB@Au were assessed by simulating the penetration of NIR lasers through the skin of different thicknesses before targeting cancerous cells (see scheme in **Figure** **7**A) and digital images Figure 7B. Before this experiment, the laser penetration capabilities of NIR‐I and NIR‐II lasers were evaluated. Results showed that the NIR‐I laser lost up to 64 mW of energy when passing through a 4 mm thickness of 0.6% agarose gel (mimicking human brain tissue).^[^ ^50^ ^]^ On the other hand, the NIR‐II laser lost up to 40 mW while passing through a 4 mm thick gel. However, both the NIR‐I and NIR‐II lasers lost nearly the same amount of power, ≈70 mW, when passing through a 6 mm thickness, possibly due to the laser power dissipation saturation at greater depths, as shown in Figure 7C.

Upon irradiation with NIR‐I and NIR‐II lasers, the BB@Au embedded gel exhibited a significant temperature increase monitored via thermal imaging (Figure 7D–F). Notably, when the transparent hydrogel was absent (0 mm thickness condition), the temperature rise was lower compared to the condition with an additional hydrogels layer on top (2–6 mm thickness conditions). This was due to the liquefaction of the BB@Au embedded hydrogel within the first 2 min of laser irradiation, which resulted in minimal temperature rise. Moreover, under conditions where the transparent gel thickness was 6 mm, all the tissue mimics exhibited a temperature rise of up to 43, 41.4, and 43.7 °C, for BB_400_@Au, BB_800_@Au, and BB_1600_@Au particles, upon irradiation with the NIR‐I laser for 5 min. Meanwhile, irradiation with the NIR‐II laser on a 6 mm thickness gel resulted in temperature increases of 48, 43.6, and 44 °C for BB_400_@Au, BB_800_@Au_,_ and BB_1600_@Au, respectively. The ability of BB@Au to induce temperature rise through different tissue depths supports their potential in controlled and precise heating of deep‐seated tumors, which further enhances their potential for clinical translation.

**Figure 8:**
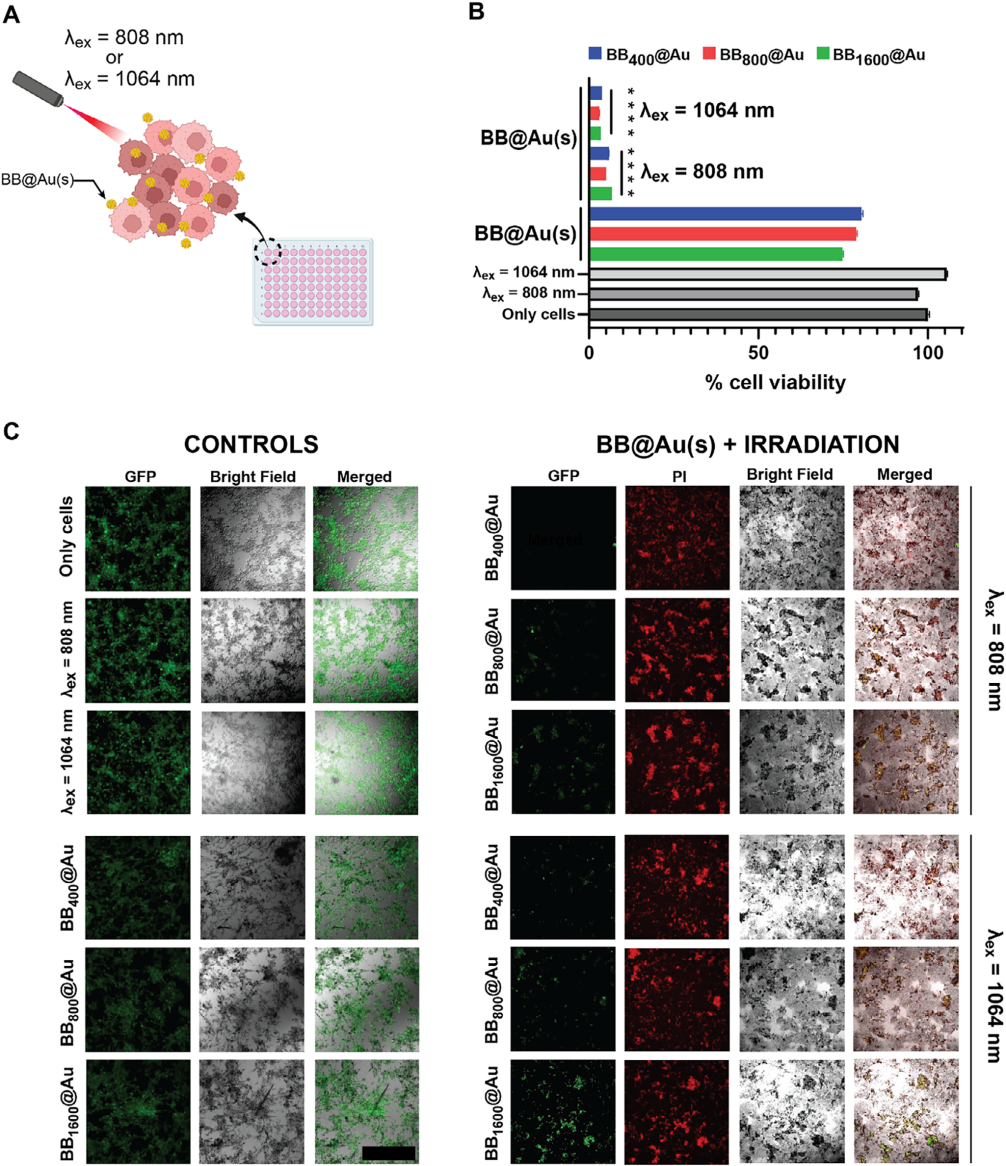
Photothermal ablation of cancer cells using BB@Au particles in 2D cell culture. A) Schematic showing the irradiation of cells in 96 well plate by NIR‐I and NIR‐II lasers; **B)** Quantitative analysis of 2D photothermal cell death induction in different groups, ^****^ *p* ≤ 0.0001; C) Bright field and fluorescence images of cells after photothermal treatment in different groups, the magnification used was 10X. Abbreviations: GFP: Green fluorescent protein channel, PI: propidium iodide channel. Data are shown as mean ± SD (*n* = 3). Statistical significance was determined by two‐way ANOVA with Tukey's post‐hoc test, with *p* < 0.05 considered significant (^****^ *p* ≤ 0.0001).

The photothermal ablation potential of BB@Au particles was evaluated on a GFP‐expressing human glioblastoma cell line (U87) in both 2D and 3D culture settings to get insights into their efficacy in causing cell death. In the 2D photothermal ablation experiments, as shown in schematic **Figure** **8**A, after NIR‐I and NIR‐II laser irradiation, nearly all conditions except controls experienced a decline in viability to less than 10%, as depicted in Figure 8B. Specifically, after treatment with BB_400_@Au, BB_800_@Au, and BB_1600_@Au in combination with NIR‐I laser, the cell viability dropped to 6.73 (±0.005)%, 5.04 (±0.016)%, and 5.88 (±0.014)%, respectively.

Similarly, under NIR‐II laser irradiation, the viability decreased down to about 3%. This reduction in viability was also evident in the qualitative analysis using propidium iodide, PI, (Figure 8C **),** where the loss of GFP fluorescence was observed in the BB@Au + NIR laser groups, while wells without irradiation showed negligible toxicity, similar to the control groups. The substantial decrease in cell viability, as evident in both quantitative and qualitative analyses, highlights the effectiveness of BB@Au in eliciting cellular damage and subsequent cell death when activated by specific NIR wavelengths. The negligible toxicity observed in control groups and wells without laser irradiation reaffirms the minimal impact of photothermal agents alone on cell viability, emphasizing their selectivity and activation by NIR light for inducing photothermal ablation.

**Figure 9:**
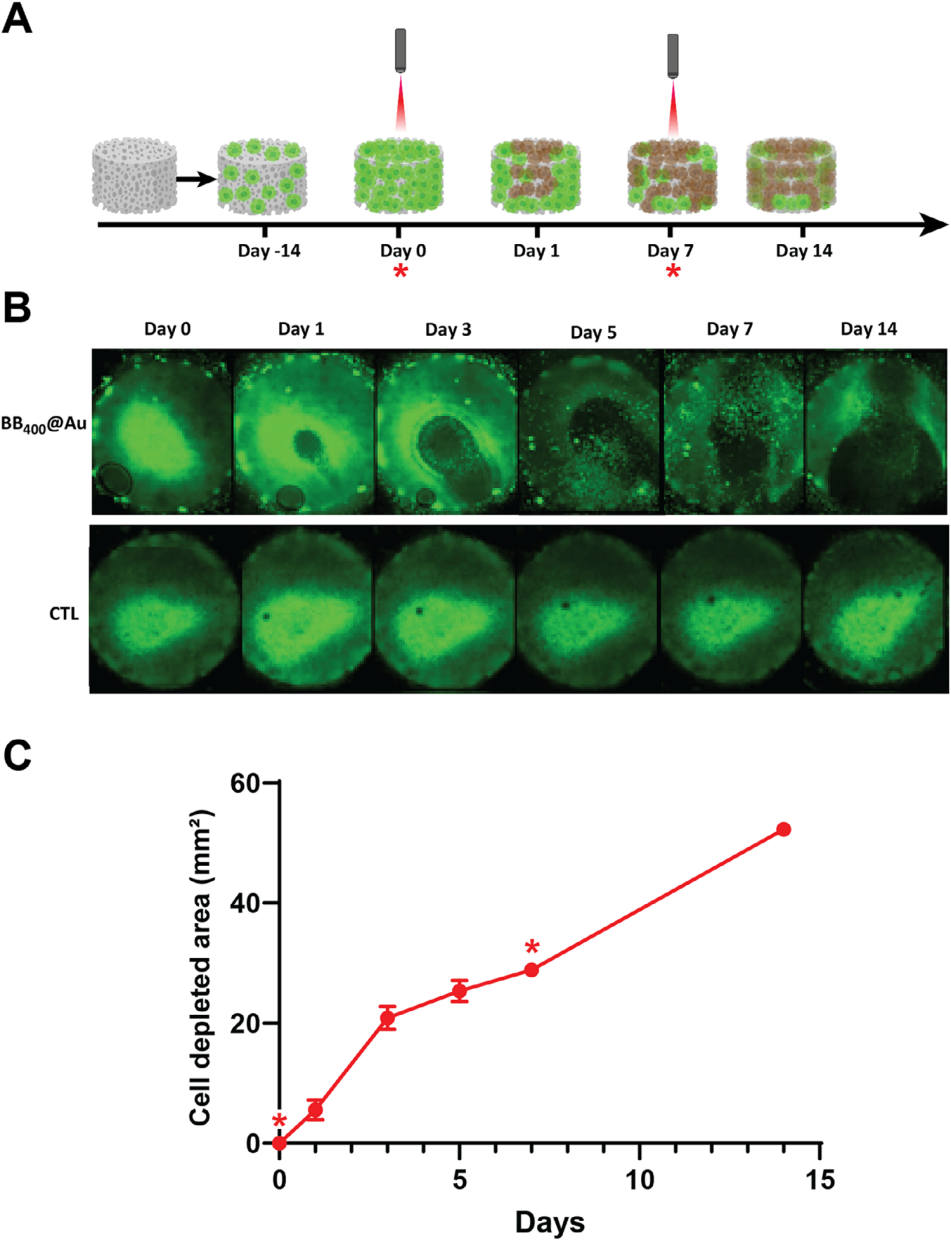
Evaluation of photothermal ablation of 3D tumor models by BB@Au particles A) The schematic showing the timeline of the treatment plan; B) Confocal images of the 3D scaffolds populated with GFP expressing U87 cells at different time points. C) The change in the area of cell depleted region after the laser irradiation of the 3D scaffold. Data are presented as mean ± SD (*n* = 3).

The therapeutic efficacy of BB_400_@Au mediated NIR‐II photothermal therapy was also examined in a 3D cell culture setting using commercially available 3D inserts, as shown in **Figure** **9**A. Fluorescence confocal imaging was employed following the addition of BB_400_@Au, and NIR‐II irradiation was conducted. Upon NIR‐II irradiation, the surface temperature of the wells treated with BB_400_@Au reached above 43 °C. Conversely, in the irradiation control (no BB@Au particle), the temperature did not exceed 37 °C (physiological range). Following treatment, the growth of the 3D tumor was monitored for 2 weeks post‐treatment. The area treated with BB_400_@Au + NIR‐II laser exhibited almost complete ablation at the laser spot and efficient inhibition of distant growth cells, as shown in Figure 9B. The inhibition zone was expanded up to the boundary of 3D inserts on 5th day after treatment, as shown in Figure 9B. On the 7th day, cancer cells started relapsing, which was controlled by a second irradiation of the NIR‐II laser at the same power and irradiation time as the first one. The second irradiation led to the expansion of the ablation zone up to 80% of the area of the 3D insert on 14th day, as observed in Figure 9B. In contrast, there was no such ablation or inhibition zone observed for the control condition during the study.

Figure 9C quantitatively captures this dynamic response by illustrating the time‐dependent change in the cell‐depleted area post‐irradiation. The area of ablation increased rapidly during the first 5 days, reaching over 25 mm^2^, followed by a slower expansion phase. After the second irradiation on day 7, the depleted area showed a pronounced increase, ultimately surpassing 50 mm^2^ by day 14. The trend indicates an initial burst of ablation due to the first dose, followed by extended spatial depletion enabled by the second treatment. These quantitative results support the qualitative imaging findings and demonstrate that BB_400_@Au‐mediated NIR‐II photothermal therapy is effective in inducing both immediate and progressive tumor cell ablation over time.

The observed initial ablation and inhibition of distant cell growth highlight the efficacy of BB@Au‐mediated photothermal therapy in controlling tumor expansion. However, the observed relapse and limited long‐term control suggest the need for refining treatment strategies, potentially involving multiple or sustained treatment cycles to achieve prolonged suppression of tumor regrowth. To further enhance therapeutic efficacy and overcome tumor recurrence, future work could explore the integration of BB@Au NPs with chemotherapeutic agents to enable combinatorial therapy. The BB polymer scaffold offers potential functionalization sites and internal volume that could be exploited for loading or conjugating chemo‐drugs, thereby enabling synergistic effects between photothermal and chemotherapeutic modalities. Such a dual‐approach may improve both primary tumor ablation and long‐term control by targeting residual tumor cells more effectively. Despite these considerations, the current findings lay a strong foundation for the continued development and optimization of BB@Au‐based platforms for real‐world solid tumor treatment.

**Figure 10:**
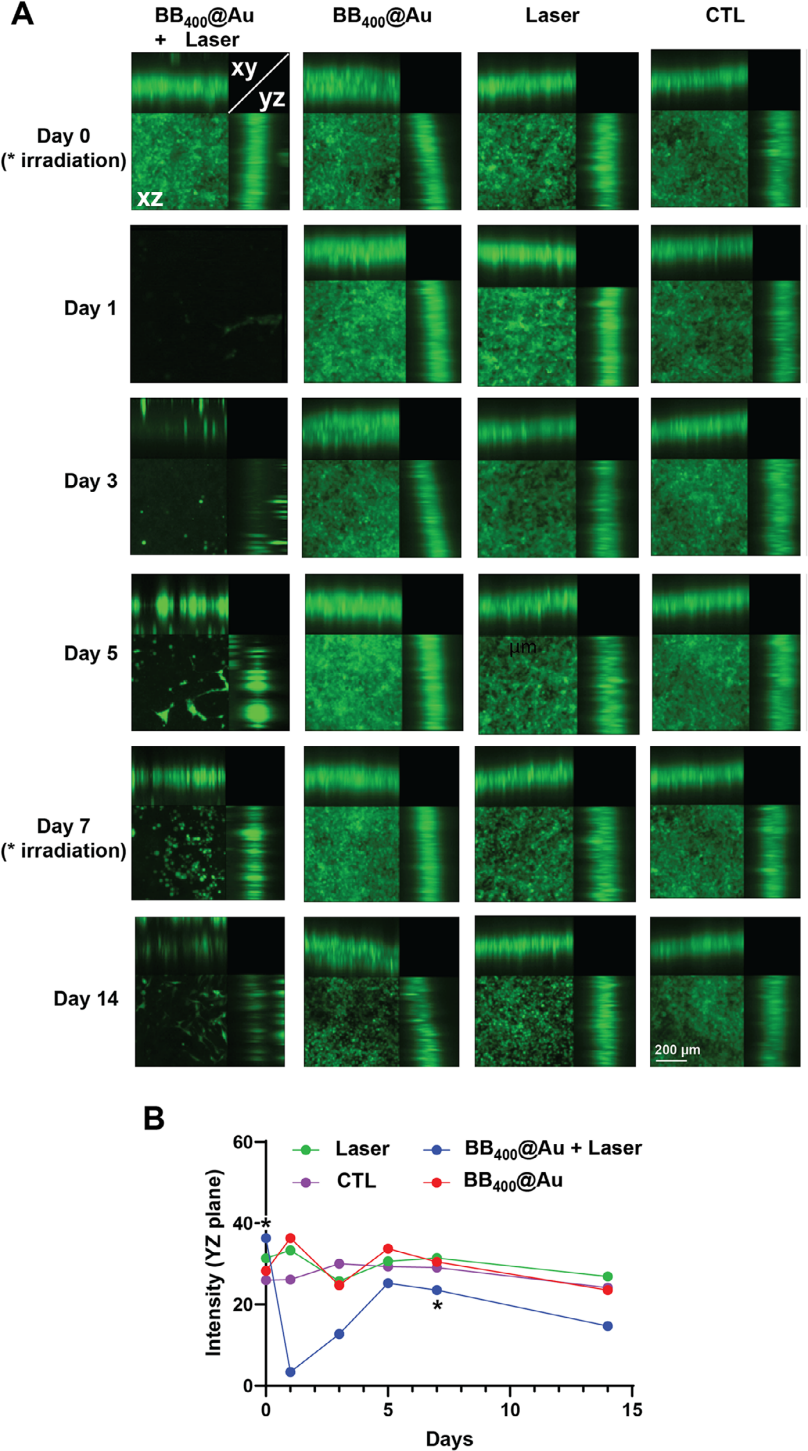
Evidence of efficient photothermal cell ablation throughout thick 3D cultured tissues mediated by BB@Au particles. A) The orthogonal projections (with maximum intensity) of 3D inserts at different time points. The laser irradiation was performed on 0th day and 7th day of the study. B) The time evolution of fluorescence intensity in the YZ projection.

Confocal microscopy was also performed to assess the depth and detailed spatial distribution of the photothermal ablation depth in the direction of irradiation, (*xz* and *yz*‐axis), as shown in **Figure** **10**A. Notably, the group treated with laser alone did not show any ablation or inhibition throughout the thickness of the scaffold. In contrast, the group treated with both BB_400_@Au and the NIR‐II laser showed clear and substantial cell ablation through the whole thickness of the scaffold. The XZ and YZ‐axis optical sectioning unveiled a distinct and considerable penetration depth of the ablation effect, indicating the successful synergistic interaction between the BB_400_@Au and the NIR‐II laser, as shown in Figure 10B. Interestingly, in the YZ‐plane intensity profile of the BB_400_@Au + laser group, a sudden drop in fluorescence intensity was observed at Day 1, followed by a partial recovery before the second irradiation. This phenomenon is likely attributable to an initial thermal ablation‐induced collapse of cells at the laser focus, which created a depleted region, followed by redistribution of live fluorescent cells from surrounding areas into the ablated zone due to cell migration and proliferation. The second irradiation halted this regrowth, leading to sustained ablation in the subsequent days. These results reinforce and add a quantitative dimension to the qualitative observations of earlier results. The depth assessment through *z*‐axis optical sectioning provides concrete evidence of the NIR‐II driven efficacy of the photothermal treatment.

The ablation efficacy of the photothermal treatment was further confirmed by histological analysis of the inserts collected at the end of the study. A distinct patch of ablation (dashed rectangle) was observed in the BB_400_@Au+ NIR‐II laser group, while no such area was evident in the control group, as shown in Figure S14 (Supporting Information).

### 2.5 Surface‐Enhanced Raman Scattering‐Based Analytes Detection

**Figure 11:**
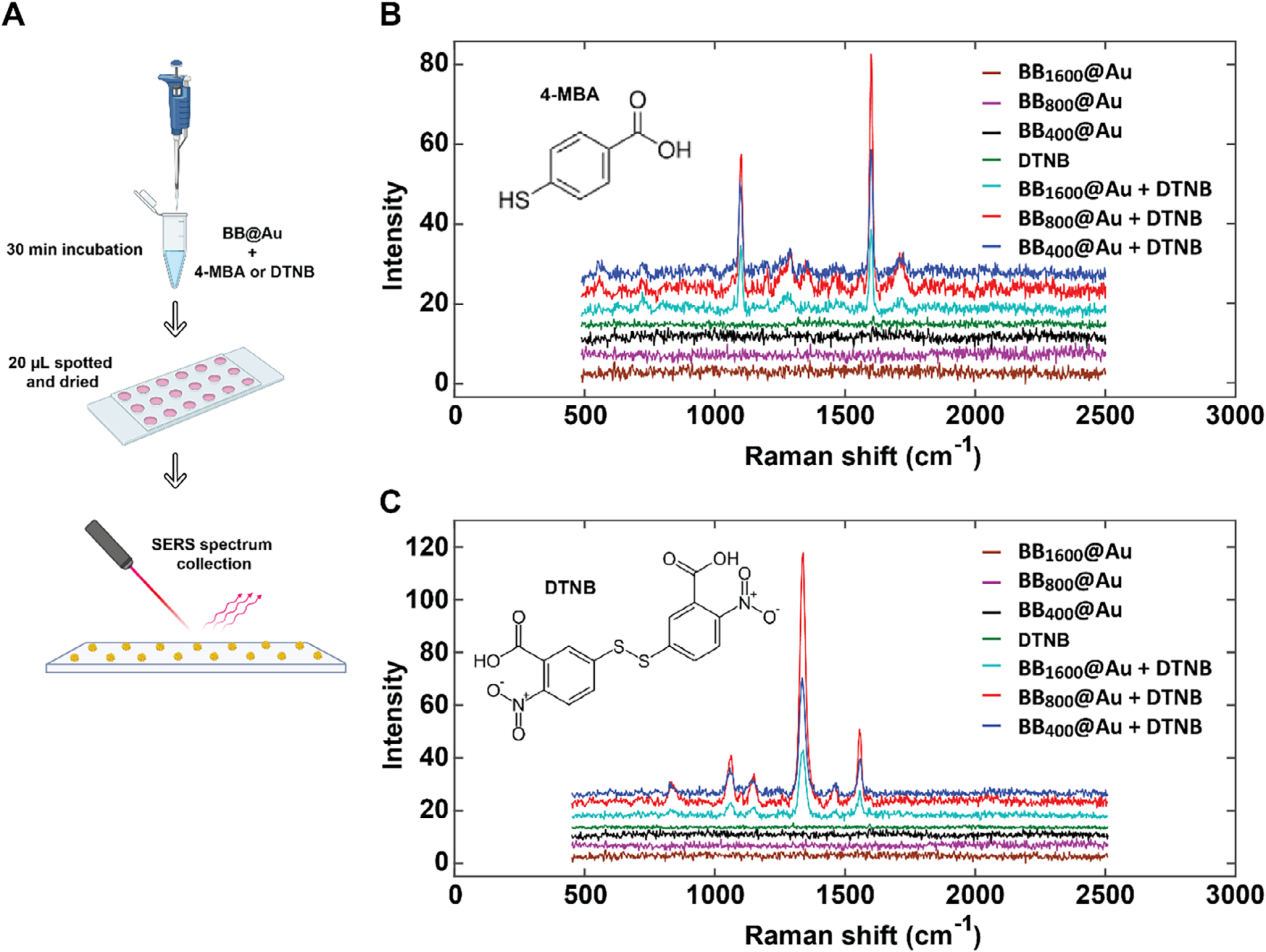
Representative SERS spectral analysis of 4‐MBA and DTNB in the presence of BB_400_@Au, BB_800_@Au, and BB_1600_@Au nanoparticles: A) schematic showing the sample preparation for SERS studies. B) SERS spectra of 4‐MBA and C) DTNB in the presence and absence of BB@Au plasmonic nanoparticles.

Leveraging on the BB@Au particles’ unique architecture, we tested their capacity to detect adsorbed analytes via Surface Enhanced Raman Scattering (SERS). Normalized concentrations of BB@Au particles were mixed with 4‐mercaptobenzoic acid (4‐MBA) and 5,5′ dithiobis(2‐nitrobenzoic acid) (DTNB) Raman reporters to a final concentration of 1 µm in separate vials (**Figure** **11**A). These two reporters were selected for SERS studies because they exhibit large scattering cross‐sections and thiolated terminal groups that bind strongly to gold nanoparticle surfaces. Thus, SERS spectra of BB@Au/4‐MBA and BB@Au/DTNB conjugates exhibited significantly enhanced spectral intensities than the Raman spectra of blank samples (i.e., BB@Au alone, 4‐MBA, and DTNB alone), which provides evidence on the influence of plasmonic BB@Au on the enhancement of Raman signals from low concentrations of 4‐MBA and DTNB adsorbed onto the surface of AuBBs particles. The characteristic peaks detected at 1078 and 1590 cm^−1^ are assigned to the aromatic ring vibrations in 4‐MBA, while bands at 1063, 1338, and 1556 cm^−1^ (Figure 11B,C) are attributed to the ring breathing modes, ─NO_2_ stretching vibrations, and aromatic ring vibrations in DTNB, respectively. The spectral features detected in this study (Figure 11) agree with those reported in the literature.^[^ ^51^, ^52^ ^]^

It is worth mentioning that BB_800_@Au yielded stronger SERS spectral signals than BB_400_@Au and BB_1600_@Au for both reporter molecules investigated in this work, perhaps due to the presence of dense and closely packed spikes on BB_800_@Au (Figure S5, Supporting Information), which potentially yielded stronger localized surface plasmon resonance (LSPR) hotspots, analogous to the electromagnetic properties exhibited by plasmonic metallic nanostars.^[^ ^53^ ^]^ To further quantify the analytical sensitivity, we tested the SERS activity of BB_800_@Au particles by detecting a series of MBA concentrations. SERS spectra for various levels of 4‐MBA are shown in Figure S14 (Supporting Information), with the corresponding linear regression plot based on the 1590 cm^−1^ band. The regression analysis (*R* ^2^ = 0.994) and extrapolation of the signal‐to‐noise ratio yielded an estimated limit of detection (LOD) of 12.9 nM, demonstrating the high analytical sensitivity of BB_800_@Au particles. These findings underscore the potential of BB@Au particles as effective enhancers for Raman scattering, and opens the spectrum of applications of these materials in areas such as sensing, imaging, and diagnostics.

## 3 Conclusions and outlook

The photothermal therapy offers a non‐invasive way to control or ablate certain cancers and relies on the heat produced by the molecular transducer. In addition to locally ablating the solid tumor, it also induces immunogenic cell death, ultimately contributing to the systemic enhanced antitumor immune response. Despite the growing interest in photothermal therapy, its translation potential has been limited due to the plague of variability in the synthesis of nanomaterials. We herein synthesized the first‐time reported bottlebrush template‐assisted facile synthesis of photothermal agents that are responsive to both NIR‐I and NIR‐II lasers, owing to superior tissue penetration for the complete ablation of solid tumor.

Development of NIR‐II photothermal agent in a facile and scale‐up way is imperative for the translation of the photothermal therapy from bench to bedside. However, most of the NIR‐II photothermal agents come from the chalcogenides whose toxicity profile is controversial, and have the issue of leaking ions that may cause chronic toxicity. In contrast, BB@Au particles offer a distinct advantage. They are produced from well‐understood polymer BB structures combined with biologically inert gold. What makes BB@Au particles particularly noteworthy is the absence of the need for synthesizing polymeric nanoparticles as a template. These characteristics bypass a common step in photothermal agents’ production, streamlining the process while utilizing materials known for their biocompatibility and stability. This streamlined approach not only enhances the efficiency of production but also reduces the potential risks associated with using chalcogenides, thus positioning BB@Au particles as promising candidates for safe and effective NIR‐II photothermal therapy.

In summary, we have developed a library of soft template‐assisted NIR‐I and NIR‐II light driven photothermal agents for photothermal ablation of cancer. This work not only illustrates the method of utilizing the soft template for the synthesis of photothermal agents but also unveils the library of other photothermal agents that could be developed simply by changing the structure of the polymer. The detailed characterization of AuBBs serves as a foundation for understanding the structural modifications induced by gold coating on the bottlebrush polymers, shedding light on their composition, morphology, and surface properties. Such insights are crucial for correlating the structural characteristics of these nanomaterials with their intended applications in therapeutics and diagnostics realms.

## 4 Experimental Section

### Materials

All chemicals were purchased from Sigma‐Aldrich unless otherwise stated. Triethylamine (TEA) was dried with calcium hydride and freshly distilled every time before use. 2‐(Trimethylsilyloxy)ethyl methacrylate (HEMA‐TMS), 2‐(dimethylamino)ethyl methacrylate (DMAEMA), methyl methacrylate (MMA) were passed through a basic Al_2_O_3_ column to remove inhibitor. Potassium fluoride, 2,6‐di‐tertbutylphenol, 2‐bromoisobutyryl bromide (BIBB), 4,4′‐dinonyl‐2,2′‐dipyridyl (dNbpy), copper (II) bromide (CuBr_2_), copper(I) bromide (CuBr), Tributyltin hydride, tetra‐*n*‐butylammonium fluoride (TBAF), potassium carbonate were used without further purification. Ethylene bis(2‐bromoisobutyrate) (2f‐BIB) was synthesized according to the following references.^[^ ^54^, ^55^ ^]^ Inserts for 3D cell culture were purchased from Alvetex. All glassware was cleaned with aqua regia before usage.

### Instrumentation

Absorbance spectra of BB@Au particles were recorded in the Shimadzu UV‐2600i spectrometer. The hydrodynamic size and zeta potential of BB@Au particles were measured by Malvern Zetasizer. TEM images were captured by Talos f200x at McGill University, Canada, and Jeol (JEM‐2100F) at Polytechnique Montreal, Canada. The photothermal temperature of the solution and 3D inserts was measured by probe thermometer (Thorlabs, TSP01) and FLIR thermal imaging camera (FLIR E6XT). The key turn laser sources NIR‐I (808 nm) & NIR‐II (1064 nm) were purchased from RPMC Lasers, USA. The dark field and bright field images were captured by Zeiss LSM 880 confocal microscope in the Institut de recherche en immunologie et en cancérologie (IRIC), Canada. The side illumination images were captured by a Nikon inverted microscope in Polytechnique Montreal, Canada. H&E images of zebrafish and 3D scaffold were captured by Zeiss CSU X1 in Dalhousie University, Canada. The NMR measurements were performed using a Bruker AV‐III 400 MHz, and the AFM measurements were conducted with a JEOL JSTM‐4200. The calorimeter measurements were performed using a plate reader (TECAN Spark)

### Synthesis of P(MMA‐*co*‐HEMA‐TMS)

The synthesis followed the previous report with slight modifications.^[^ ^56^, ^57^, ^58^ ^]^ The atom transfer radical polymerization (ATRP) method was used to obtain the three block copolymers with different backbone units. The polymerization procedures were the same, except for slight variations in reaction time. As an example, the synthesis of ‐P(MMA‐*co*‐HEMA‐TMS) (BB_400_) was conducted as follows. A dry 10 mL Schlenk flask was charged with 2f‐BIB (4.1 mg, 11.3 µmol), CuBr_2_ (3.0 mg, 13.5 µmol), dNbpy (66.3 mg, 162.3 µmol), HEMA‐TMS (2.3 g, 11.3 mmol), MMA (1.1 g, 11.3 mmol), and anisole (1.0 mL). The solution was degassed by three freeze‐thaw cycles. During the final cycle, the flask was purged with argon, and CuBr (7.8 mg, 54.1 µmol) was quickly added to the frozen reaction mixture. The flask was sealed, evacuated, and purged with nitrogen five times, and then immersed in an oil bath at 40 °C. After 3 h reaction, a small amount of the reaction mixture was taken for NMR to check the conversion ratio of the monomers. Tributyltin hydride (9.8 µL, 33.8 µmol) was added to the reaction mixture and let the reaction continued for another 2 h. Then, the reaction was stopped via exposure to air. The monomer conversion ratio was calculated by the integration of MMA and HEMA‐TMS, vinyl groups signal (*CHH═*C─CH_3_, *δ* = 6.11 ppm or *δ* = 5.56 ppm) against the hydrogen of the backbone (─CH_2_─C(*CH_3_*)‐, *δ* = 0.75‐1.0 ppm). The polymer was purified by precipitating the solution into cold methanol twice (cooled with dry ice and isopropanol). The product was redissolved in CHCl_3_ and passed through a short natural Al_2_O_3_ column. After evaporating most of the solvent, the polymer was dried under vacuum for 16 h at room temperature. 10 mg of dried polymer was taken out for recording the ^1^H NMR spectroscopy.

### Synthesis of Macroinitiator P(MMA‐*co*‐PBiBEM)

The synthesized P(MMA‐*co*‐HEMA‐TMS) was first hydrolyzed to P(MMA‐*co*‐HEMA). A typical procedure was followed. Briefly, the polymer (0.4 g, 6.2 µmol, 1.3 mmol HEMA‐TMS moieties) was dried and redissolved in 10 mL dimethylformamide (DMF). Potassium fluoride (114.8 mg, 1.9 mmol) was added to the reaction mixture, followed by the dropwise addition of 1.6 mL (1.6 mmol) of tetra‐*n*‐butylammonium fluoride (TBAF). The mixture was then stirred for 24 h. The mixture was then dialyzed against DMF and water mixture (v/v 1:1) (distilled water containing two drops of HCl) with Spectrum Spectra/Por RC Dialysis Membrane (Molecular weight cut‐off 50K). The polymer was then dialyzed against acetone. After evaporation of the solvent, the polymer was dried under vacuum. The polymer was transferred to a round bottom flask, and 547 mg K_2_CO_3_ (3.9 mmol) was added to the flask. The polymer was then redissolved in 10 mL dry DMF, and the solution was cooled to 0 °C. 0.33 mL 2‐bromoisobutyryl bromide (2.6 mmol) was added dropwise into the solution. The reaction was continued overnight. After the reaction was done, the solid salt was centrifuged off. The liquid was dialyzed against DMF/H_2_O, acetone/H_2_O, and then acetone. The acetone was evaporated, and the polymer was redissolved in CHCl_3_, and passed through basic Al_2_O_3_. The solution was concentrated and precipitated into hexane for three times. After drying in the oven, the purified polymer was analyzed with and kept in −80 °C.

### Polymerization of DMAEMA ((poly((methyl methacrylate)‐*co*‐(2‐(2‐bromoisobutyryloxy)ethyl methacrylate))‐*g*‐poly(2‐(dimethylamino)ethyl methacrylate) (P(MMA‐*co*‐BiBEM)_418_‐*g*‐PDMAEMA)_76_)

A dry 10 mL bottom round flask was charged with CuBr (2.5 mg, 17.6 µmol)), CuBr_2_ (0.44 mg, 1.9 µmol), and dNbpy (15.9 mg, 39.1 µmol). The flask was sealed with a stopper and black tape. In the meantime, BB‐Br macroinitiator (10 mg, 25.6 µmol) and DMAEMA (922.5 mg, 5.9 mmol) were dissolved in anisole (2.0 mL) under stirring for a few minutes in a separate vial. A stainless‐steel cannula was used to connect the flask to the vial. Then, the solution (in the vial) was degassed by four freeze‐pump‐thaw cycles. During the final cycle, the flask was thawed to room temperature and filled with nitrogen. The whole system was maintained at a vacuum for a few seconds, and then the cannula was inserted into the solution. The system was quickly switched to argon to push the solution from the vial to the flask. The flask was sealed tightly after the removal of the cannula. The mixture was put in a pre‐heated oil bath at 50 °C. Aliquot samples were taken for NMR to check the monomer conversion ratio. The reaction was stopped via exposure to air. The polymer was purified by dialysis against DMF and acetone mixture (v/v = 1/1), acetone alone, and dried under vacuum.

### Synthesis of Final Bottle Brush Polymer P(MMA‐*co*‐BiBEM)_418_‐*g*‐qPDMAEMA)_76_

Quantity of 0.2 g (0.082 µmol, 1.23 mmol PDMAEMA moieties) synthesized P(MMA‐*co*‐BiBEM)_418_‐*g*‐PDMAEMA_76_ was dissolved with 10 mL acetone in a round bottom flask. The solution was cooled to 0 °C with an ice bath. Then, 0.5 mL of 2‐bromoethane (6.8 mmol) was added slowly into the solution. The reaction was continued for 48 h at room temperature. The polymer solution was dialyzed against methanol to remove the impurity. After dialysis, it was stored in −20 °C till usage.

### Synthesis of Gold Deposited BB (BB@Au NPs)

The gold was deposited on the BB polymers of different unit lengths following the in‐situ method as described previously.^[^ ^33^, ^34^ ^]^ Briefly, the solvent of BB polymer was replaced with water using the rotavapor operating at 100 mm Hg. The BB was redissolved in water to prepare the 1 mg mL^−1^ stock. In a 1 mL clean glass vial, 100 µL of BB was stirred at moderate speed, and then 5 µL of ascorbic acid (25 mm), which served as a reducing agent, was added dropwise. The mixture was left stirring for 2 min then, the different volumes of HAuCl_4_ (10 mm), which acts as the source of gold ions, were added and left to stir for another 2 min to promote interaction and even deposition. The BB@Au NPs were pelleted down using the swing rotor centrifuge at 5000 g for 10 min. After that, it was redispersed in the same volume of water, and absorbance was recorded using the UV−vis–NIR spectrophotometer.

### Stability Studies of BB@Au NPs

The stability studies of BB@Au NPs were performed by incubating them for different time periods. The 1 mL of 1 mg mL^−1^ BB@Au NPs in water were incubated in clean 5 ml glass vials for 2, 4, and 7 days at room temperature daylight without stirring. After that, the absorbance, hydrodynamic diameter, and zeta potential measurements were recorded and analyzed at each time point. Any shifts in absorbance spectra, changes in particle size, or fluctuations in surface charge were noted.

### Folding of BB@Au NPs

The folding of BB@Au NPs was determined with the help of elemental mapping of carbon, nitrogen, and gold. Briefly, 5 µL of BB_400_@Au_,_ BB_800_@Au_,_ and BB_1600_@Au at the concentration. of 1 µg mL^−1^ were drop cast on the TEM copper grids. These solutions were evenly spread to ensure uniform dispersion. Subsequently, the grids were left to air dry overnight at room temperature in a dark environment. The next day, elemental mapping was performed using Talos F200X. This technique involved scanning the samples with electron beams to visualize and quantify the distribution of carbon, nitrogen, and gold atoms within the AuBBs.

### Biocompatibility of AuBBs

The biocompatibility evaluation of AuBBs was performed on the human brain microvasculature cell line (hCMEC/D3) as well as zebrafish larvae. To determine the biocompatibility of AuBBs in a concentration‐dependent style, 96‐well flat bottom plates were coated with 0.33 µg cm^−2^ of collagen (dispersed in water) and incubated for 3 h. The supernatant was discarded, and wells were rinsed 2 times with phosphate‐buffered saline (PBS). After that, hCMEC/D3 cells were seeded at a density of 10^4^ cells per well, and the plate was incubated overnight for attachment. The next day, the media of the wells were replaced with 100 µL of AuBBs with different concentrations (dispersed in 100 µL of Dulbecco's Modified Eagle Medium (DMEM) complete media), ranging from 15 µg per well to 60 µg per well. After 24 h of incubation at 37 °C and 5% CO_2_, the cells were imaged using microscope at 10 X. The culture media from the wells were replaced with 100 µL of 5% 3‐(4,5‐Dimethylthiazol‐2‐yl)‐2,5‐diphenyltetrazolium bromide (MTT) dye (dispersed in 1X PBS). The plate was incubated for another 5 h in the same conditions. The supernatant was removed, and crystals were solubilized in 200 µL of Dimethyl Sulfoxide (DMSO). To avoid the overflow of the readings in the plate reader, it was further diluted 10 times before doing the colorimetric absorbance measurement at 570 and 690 nm with 10 s of shaking. The percentage viability of the cells was calculated using the following equation.

(1) $$\%\;\mathrm{cell}\;\mathrm{viability}=\frac{absorbance\;\left(570-690\right)of\;treated\;cells}{bsorbance\;\left(570-690\right)of\;untreated\;cells}\times 100$$

To determine the biocompatibility of AuBBs in time dependent manner, 12‐well flat bottom plates were coated with collagen as described previously. The hCMEC/D3 cells were seeded at 10^4^ cells per well, then serially diluted to 1:3:7 dilutions as described previously,^[^ ^6^ ^]^ and plates were left overnight in the incubator. The next day, 500 µL of AuBBs was added at a concentration of 60 µg per well in DMEM complete media. The plate was incubated, and images were captured on 1st day, 3rd, and 7th day using a bright field microscope. After imaging each day, 500 µL of 5% MTT dye in PBS was added, and after 5 h of incubation, the supernatant was removed. The crystals of all different days of incubation were dissolved together on the 7th day using 500 µL of DMSO, and colorimetric recording was done as described earlier.

The biocompatibility of AuBBs was further tested on the wild‐type zebrafish larvae (*Danio rerio*, Tupfel long‐fin (TL). The zebrafishes were maintained at a 12 h day‐night cycle in the animal facility at the Centre National de Biologie Experimentale (CNBE), Laval, Canada. They were bred as described previously, following the standard procedure.^[^ ^59^ ^]^ All experiments were completed in compliance with the local ethical committee as well as the Canadian Council for Animal Care. The eggs were collected and transferred to a Petri dish maintained at 28 °C. The 1‐phenyl 2‐thiourea (PTU, < 0.3%) was added to prevent pigmentation. The next day, the rotten eggs were discarded after checking them using microscope, and others were placed in 12‐well plates in the number of 20/well, and 100 µL of BB_400_@Au, BB_800_@Au_,_ and BB_1600_@Au were added at a concentration of 60 µg/well. The fish water was renewed after 48 h of incubation along with the fresh addition of the same formulation. The hatching and survival rates were checked every 24 h using the microscope, and pericardial edema and morphological abnormalities were noted. The experiment was performed on a total of 60 embryos for each group, and three different AuBBs formulations were tested. After 5 days of incubation, the zebrafish larvae were euthanized following the ethical guidelines.^[^ ^60^ ^]^ After that, 1 mL of 4% paraformaldehyde (PFA) was added, and zebrafish larvae were kept at 4 °C overnight. The next day, it was given for histological sectioning. The 4 µm sections of zebrafish larvae were cut and mounted on the glass slide. It was further stained with H&E following the standard procedure.^[^ ^61^ ^]^

### Side Illumination of AuBBs

The AuBBs were further characterized by side illumination microscopy (SIM, RGB LED array::2.8 × 0.35 × 0.85, Citizen CL246) by putting 100 µL of AuBBs (BB conc. 10 µg mL^−1^) on the glass slides and drying with nitrogen. Before it, glass slides were cleaned by putting in ethanol and sonicated for 30 min after that dried with nitrogen. The glass slides were further cleaned with plasma at 0.3 mBar and at full RF for 2 min. The dried AuBBs on glass slides were also covered with oil and 0.17 mm coverslip before analyzing using a side‐illumination microscopy adaptor as described previously.^[^ ^62^ ^]^ The whole zebrafish larvae as well as the longitudinal section of zebrafish larvae after H&E staining, were also analyzed by SIM by putting the sample on a glass slide and covering it up with oil and cover slip. For analyzing the whole zebrafish larvae, the glass slide with a circular concave cavity was used.

### Photo‐Transduction of AuBBs

The phototransduction potential of AuBBs was evaluated using both NIR‐I and NIR‐II lasers. The whole experiment was done in a closed dark room. Before starting the experiment, the lasers were re‐calibrated using the power meter (Thorlabs PM100D) to obtain the 500 mW cm^−2^ at the target. To determine the optimum concentration of gold, 100 µL of BB and 5 µL of ascorbic acid were put in the different wells of 96‐well plates, and after mixing them using pipette, different volumes (5 to 100 µL) of gold at 10 mm was added. After 2 min of addition, the absorbance was recorded in the plate reader. These samples were also irradiated using the NIR‐I and NIR‐II laser for 5 min, and the maximum temperature was recorded using probe thermometer. Also, 300 µL of AuBBs were put in a 1.5 mL Eppendorf tube, and NIR‐lasers were irradiated from the top of the tube for different time periods. The temperature was recorded, and images were captured using a thermal imaging camera at different time points.

### Brain Tissue Isotype Photothermal Therapy

The deep tissue penetration ability of AuBBs was evaluated by NIR‐I and NIR‐II laser by detecting the residual laser energy after brain tissue isotype (0.6% agarose gel) of different thicknesses 0, 2, 4, 6 mm was prepared and used as a model for the biological tissues. Then, the deep tissue photothermal ability of AuBBs in NIR‐I and NIR‐II windows was assessed by irradiating the lasers at 500 mW cm^−2,^ placing the different thickness transparent gel and AuBBs embedded gel together. For embedding the AuBBs in gel, 100 µL of it was added to the melted gel, and then, it was solidified by keeping it at room temperature for 3 h.

### Photothermal Therapy in 2D Settings

The human glioblastoma cells (U87) were seeded at a density of 10^6^ cells per well in 96‐well plates. After the overnight incubation at 5% CO_2_ and 37 °C, the cells checked for their attachment. The wells were divided into following groups: 1) cells only; 2) cells + NIR‐I L (laser); 2) cells + NIR‐II L; 3) cells + BB_400_@Au; 4) Cells + BB_800_@Au; 5) Cells + BB_1600_@Au; 6) Cells + BB_400_@Au + NIR‐I L; 7) Cells + BB_800_@Au + NIR‐I L; 8) Cells + BB_1600_@Au + NIR‐I L; 9) Cells + BB_400_@Au + NIR‐II L; 10) Cells + BB_800_@Au + NIR‐II L; 11) Cells + BB_1600_@Au + NIR‐II L. The AuBBs were added to the wells, and the lasers were shined for 3 min at 500 mW cm^−2,^ in the dark room. The plates were kept back in the incubator overnight. The next day, it was analyzed by confocal microscopy at 10 X both the bright field and dark field, for the qualitative analysis using the propidium iodide at concentration 1 µg mL^−1^ for staining the dead cells. The propidium iodide was added after taking out the media and washing it one time with PBS. The propidium iodide in PBS was added, and plates were left in the incubator for 15 min before capturing images using a confocal microscope at 10 X in both bright and dark field modes. The fluorescence images were captured at excitation / emission 490 nm / 510 nm for GFP and 488 nm / 610 nm for propidium iodide. The MTT assay was performed using the 5% MTT dye and 4 h incubation at 37 °C. The crystals were dissolved in 200 µL of DMSO by incubating it for 15 min before taking the calorimetric readings at 576 and 693 nm.

### Photothermal Therapy in 3D Settings

The photothermal therapy was also performed in a 3D setting using the commercially available Alvetex scaffold. Briefly, the scaffolds were activated by dipping it in 70% ethanol and then kept in 12‐well plates. It was flushed with PBS two times and DMEM media one time. After that, 1 × 10^6^ U87 cells (GFP fluorescent) in the volume of 150 µL complete DMEM media were added on the insert, and the plates were kept back in the incubator for 2 h. After the cells had been attached, the wells were flooded with 3 mL of media. The media was changed every other day, up to 10 days, and then every day. On the 15th day after cell seeding, the confluency of the inserts was determined using the confocal microscope. The 3D photothermal experiment was started on the confluent inserts as following: The inserts were divided into the following groups. 1) BB_400_@Au + NIR‐II L; 2) Only BB_400_@Au; 3) Only NIR‐II L; 4) No treatment. Before starting the photothermal therapy, the media from all wells were removed, and the 1st and 2nd group was replaced with 100 µL of complete media containing BB_400_@Au (BB conc. 1 mg/ml), prepared as earlier described, and the other groups were replaced with 100 µL of complete DMEM media. After that, plates were incubated at 5% CO_2_ and 37 °C for 1 h, and then filled the wells of all groups with 4 mL of complete media. The NIR‐II laser was irradiated for 5 min at 500 mW cm^−2^ at the center of the insert, and then the plates were kept in the incubator for 12 h. The next day, confocal imaging along with Z‐stacking and tile programming was performed at the following settings: number of tiles 5 X 5, overlap percentage 20%, Z‐stack of 30 planes with 1 µm interval. The imaging was performed on 1st, 3rd, 5th, 7th, 14th, 21st, and 28th day of the study, and media in each well was changed every other day. On the 7th day of the study, the second dose of NIR‐II laser irradiation was specifically performed on groups 1 and 3. At the end of the study, the media was aspirated from all wells and washed with PBS twice. Then, inserts were transferred to another fresh 12‐well plates and 5 mL of 4% PFA added to the wells and plates were kept at 4 °C for 12 h. Next day, PFA was aspirated, and inserts were rinsed with 4 mL of PBS thrice. The PBS was removed, and 70% ethanol was added before inserts were given for 4 µm thick sectioning and H&E staining.

### Surface‐Enhanced Raman scattering (SERS) Spectroscopy

A 4‐mercaptobenzoic acid (4‐MBA) and 5,5′ dithiobis(2‐nitrobenzoic acid) (DTNB) of 10 µL were mixed with 200 µL of AuBBs suspensions to a final concentration of 1 µm of the Raman reporters and vortexed for 2 s. The AuBBs‐reporter conjugates were then incubated for 30 min to allow for sufficient interaction time for the conjugates. Next, 15 µL of each sample and blanks were separately spotted onto a clean aluminum foil‐coated glass slide and dried at room temperature. SERS spectra were recorded using an Alpha300 WITec Raman microscope (WITec, Germany) equipped with a 632.8 nm HeNe laser, grating of 300 grooves/mm, and CCD camera. A 50 X magnifying air objective was used to view and illuminate the samples and to collect back‐scattered photons. To analyze the SERS signals, the laser power on the sample was adjusted to ≈10 mW (1 µm beam diameter) on randomly selected points on each sample, with an exposure time of 5 s for three accumulations to obtain an averaged signal of optimal signal‐to‐noise ratio. Instrumental control and data processing were performed using the Control 5 software. Sample preparation and spectral analysis were conducted under the same experimental conditions. SERS spectral data were exported and preprocessed (baseline removal using asymmetric least squares, averaged, and spectral overlay) in MATLAB software 2022b (The MathWorks Inc., Natwick, USA).

### Statistical Analysis

The statistical analysis was performed using GraphPad Prism (version 8.0.2). Data are presented as mean ± standard deviation (SD) unless otherwise noted. For comparisons between two groups, a two‐tailed unpaired Student's *t*‐test was applied, while comparisons across more than two groups were performed using one‐way ANOVA, followed by Tukey's post‐hoc test when appropriate. The threshold for statistical significance was set at *p* < 0.05. The sample size (n) for each experiment is indicated in the corresponding figure legends. All data were checked for normal distribution and homogeneity of variance prior to statistical testing, and no data points were excluded as outliers.

## Conflict of Interest

The authors declare no conflict of interest.

## Supporting information



Supporting Information

## Data Availability

The data that support the findings of this study are available from the corresponding author upon reasonable request.
