# Supplementary material for: Bottlebrush Polymer Templates for the Synthesis of Gold Nanostructures and their Applications as Photothermal Agents and SERS Substrates
Source: Small Methods. 2025 Oct 5;9(11):e01059. doi: 10.1002/smtd.202501059 (PMC12641368; doi:10.1002/smtd.202501059)
Supplement: Supplementary file 1 — Supporting Information [file SMTD-9-e01059-s001.docx]

**Supporting Information**

# **Bottlebrush polymer templates for the synthesis of gold nanostructures and their applications as photothermal agents and SERS substrates**

# Deepak S. Chauhan^1,2,3^*, Hu Zhang^1^, Jordan Robert^1^, Malama Chisanga^4^, Jean Michel Rabanel^1^, Dikran Mekhjian^1^, Charlotte Zaouter^5^, Quoc Thang Phan^1^, Wojciech Raj^1^, Sergiy Patskovsky^5^, Shunmoogum A. Patten^6^, Éric Samarut^7^, Jean-François Masson^4^, Xavier Banquy^1, 8, 9^*

^1^ Faculty of Pharmacy, Université de Montréal, Montréal, Québec H3T 1J4, Canada

^2^ Department of Microbiology and Immunology, Dalhousie University, Halifax, NS, Canada

^3^ Department of Pediatrics, IWK Research Center, Halifax, NS, Canada

^4^ Department of Chemistry, Institut Courtois, Québec Centre for Advanced Materials (QCAM), Regroupement Québécois sur les Matériaux de Pointe (RQMP), and Centre Inter disciplinaire de Recherche sur le Cerveau et l’Apprentissage (CIRCA), Université de Montréal, Montréal, Québec H3C 3J7, Canada

^5^ INRS Centre Armand-Frappier Santé Biotechnologie, Laval, Québec, H7V 1B7, Canada

^6^ Department of Engineering Physics, Polytechnique Montréal, Montréal, Québec H3T 1J4, Canada

^7^ Centre de Recherche du Centre Hospitalier de l’Université de Montréal, Department of Neuroscience, Faculty of Medecine, Université de Montréal, Montréal, Québec H3T 1J4, Canada;

^8^ Institute of Biomedical Engineering, Faculty of Medicine, Université de Montréal, Montréal H3T 1J4, Québec, Canada

^9^ Department of Chemistry, Faculty of Arts and Science, Université de Montréal, Montréal H3T 1J4, Québec, Canada

Corresponding authors: [xavier.banquy@umontreal.ca](mailto:xavier.banquy@umontreal.ca); [Deepak.chauhan@dal.ca](mailto:Deepak.chauhan@udal.ca)

**Table of Content**

1- Synthesis of the Bottlebrush polymer templates……………………………………………2

2- Preparation of gold particles from bottlebrush templates…………………………………10

3- Evaluation of biocompatibility…………………………………………………………….12

4- Evaluation of photothermal properties…………………………………………………….16

1. **Synthesis of the Bottlebrush polymer templates**

**Scheme S1:** Synthesis scheme of the different bottlebrush polymers used as templates for the production of the photothermal agents

**Table S1** Polymerization parameters of BB backbone

|  | Mole ratio of (m+n)/Br in initiator | Conversion ratio | m | n | Total backbone units |
| --- | --- | --- | --- | --- | --- |
| P(MMA-*co*-HEMA-TMS)_418_ | 1000 | 19.5% | 110 | 99 | 418 |
| P(MMA-*co*-HEMA-TMS)_880_ | 2000 | 21.5% | 231 | 209 | 880 |
| P(MMA-*co*-HEMA-TMS)_1636_ | 2000 | 40.9% | 425 | 393 | 1636 |

**m**: repeating unit number of MMA; **n**: repeating unit number of HEMA-TMS

**Fig. S1.** ^1^H NMR spectrum of a representative BB backbone polymer, P(MMA-*co*-HEMA-TMS)_418_ recorded in CDCl_3_.

**Fig. S2.** ^1^H NMR spectrum of a representative BB macroinitiator, **P(MMA-*co*-BiBEM)_418_** recorded in CDCl_3_.

**Table S2.** Polymerization of pendent chain, PDMAEMA

|  | Mole ratio of x/Br | Conversation ratio | x | m | n |
| --- | --- | --- | --- | --- | --- |
| P(MMA-*co*-BiBEM)_418_-*g*-PDMAEMA_76_ | 228 | 33.6 % | 76 | 110 | 99 |
| P(MMA-*co*-BiBEM)_880_-*g*-PDMAEMA_60_ | 228 | 26.4 % | 60 | 231 | 209 |
| P(MMA-*co*-BiBEM)_1636_-*g*PDMAEMA_90_ | 228 | 39.5 % | 90 | 425 | 393 |

**m**: repeating unit number of MMA; **n**: repeating unit number of HEMA-TMS; **x**: repeating unit number of PDMAEMA

**Fig. S3.** ^1^H NMR spectrum of a representative non- quaternized polymer, **P(MMA-*co*-BiBEM)_418_-*g*-PDMAEMA_76_** recorded in D_2_O.

**Table S3** Summary of the final quaternized bottle brush polymers

|  |  | DP_BB_ | Grafting density | DPsc | Total M_w_ (g/mol) |
| --- | --- | --- | --- | --- | --- |
| BB_400_ | **P(MMA-*co*-BiBEM)_418_-*g*-qPDMAEMA_76_** | 418 | 47.3% | 76 | 4,083,046.00 |
| BB_800_ | **P(MMA-*co*-BiBEM)_880_-*g*-qPDMAEMA_60_** | 880 | 47.5% | 60 | 6,838,948.00 |
| BB_1600_ | **P(MMA-*co*-BiBEM)_1636_-*g*-qPDMAEMA_90_** | 1636 | 48.0% | 90 | 19,134,783.00 |

**DP**: total backbone units; **Grafting density**: the number of end-tethered qPDMAEMA polymer chains divided by total backbone units; **DPsc**: polymerization degree of side chain qPDMAEMA; **Total M_w_ (g/mol)**: the total molecular weight of the BB polymers.

**Fig. S4.** ^1^H NMR spectrum of a representative quaternized polymer, **P(MMA-*co*-BiBEM)_418_-*g*-qPDMAEMA_76_** recorded in D_2_O.

1. **Preparation of gold particles from bottlebrush templates**

**
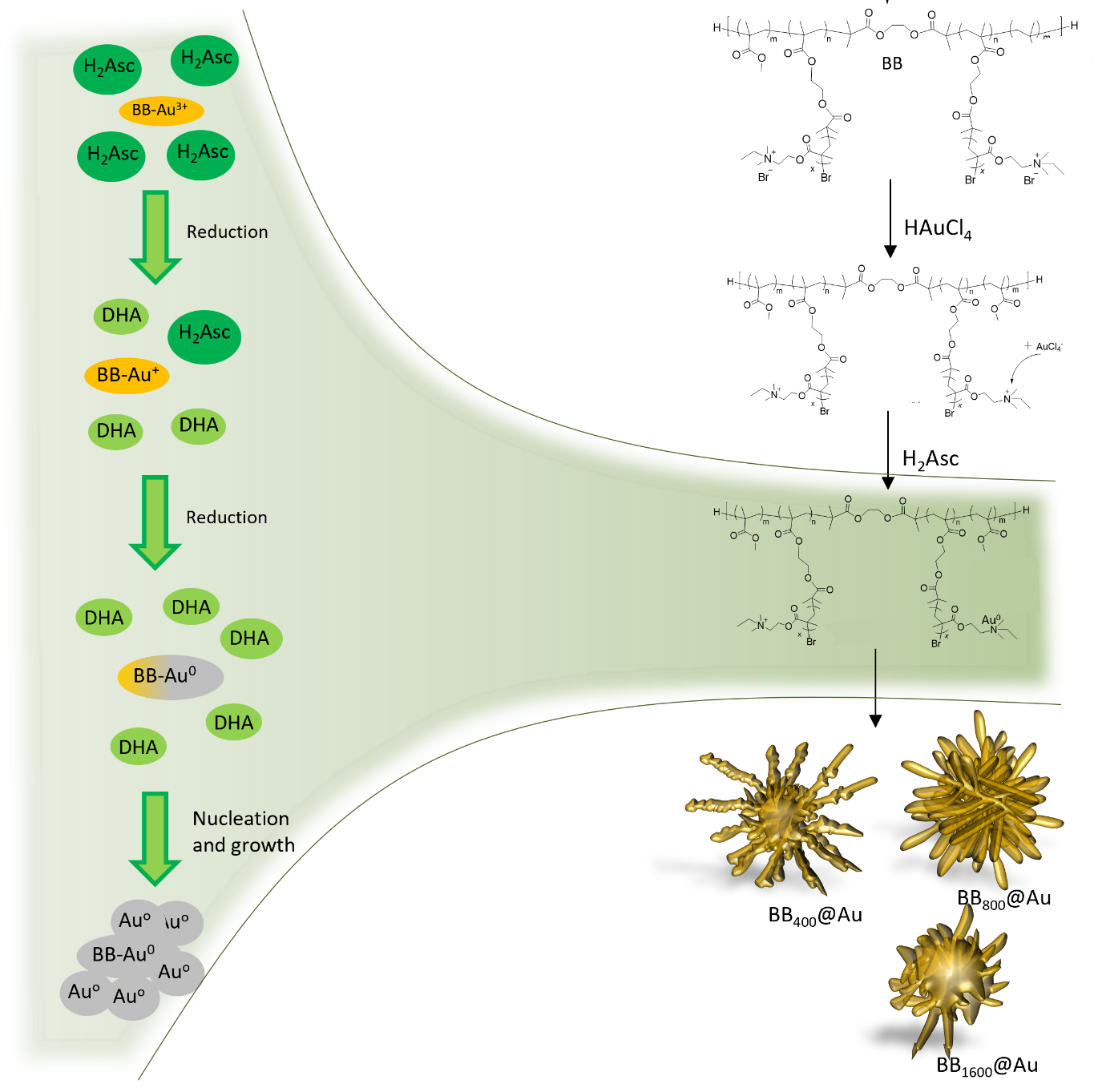
**

**Fig. S5** **Synthesis pathway of BB_400_@Au, BB_800_@Au and BB_1600_@Au.** Abbreviations: BB: Bottlebrush polymer (BB-qPDMAEMA); BB_400_@Au, BB_800_@Au and BB_1600_@Au: Gold deposited BB backbone repeating units of 418, 880, and 1636, respectively; H_2_Asc: Ascorbic acid; DHA: Dehydroascorbic acid; HauCl_4_: Chloroauric acid; BBAu^3+^: Gold BB ion in 3+ oxidation state; BBAu^3+^: Gold BB ion in 1+ oxidation state; BBAu: Gold BB in nucleation stage.


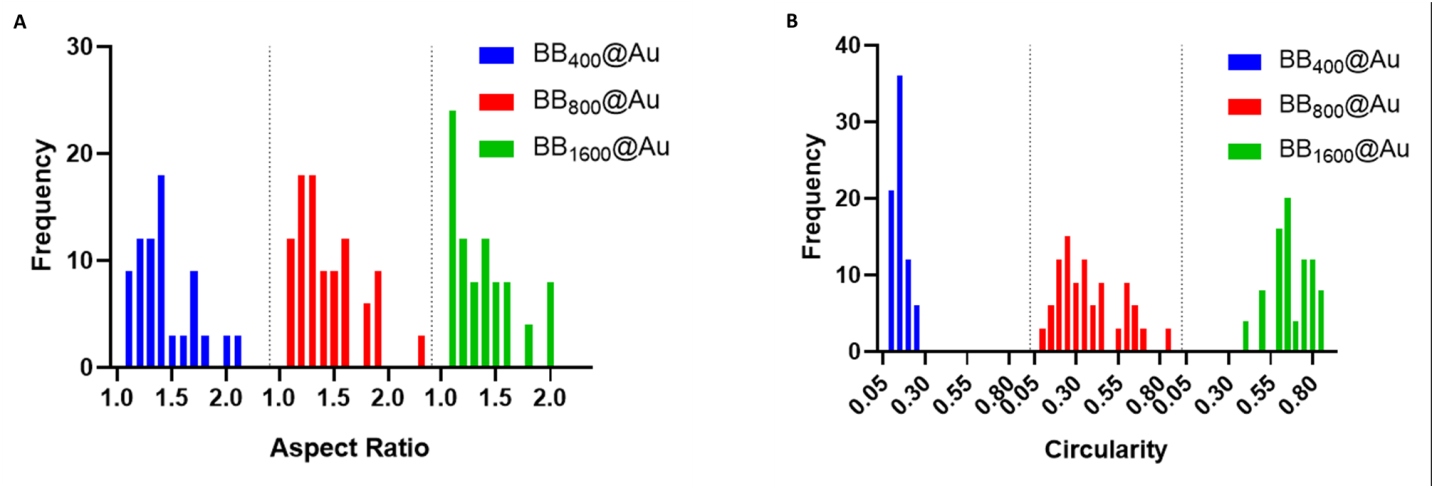


**Fig. S6.** Aspect ratio (left) and circularity (right) distributions of BB_400_@Au (blue), BB_800_@Au (red), and BB_1600_@Au (green). BB_400_@Au shows broader aspect ratio values and lower circula6rity, indicating more irregular and anisotropic particle geometries. In contrast, BB_800_@Au and BB_1600_@Au exhibit narrower distributions with higher circularity values, showing closeness to spherical geometry.

**
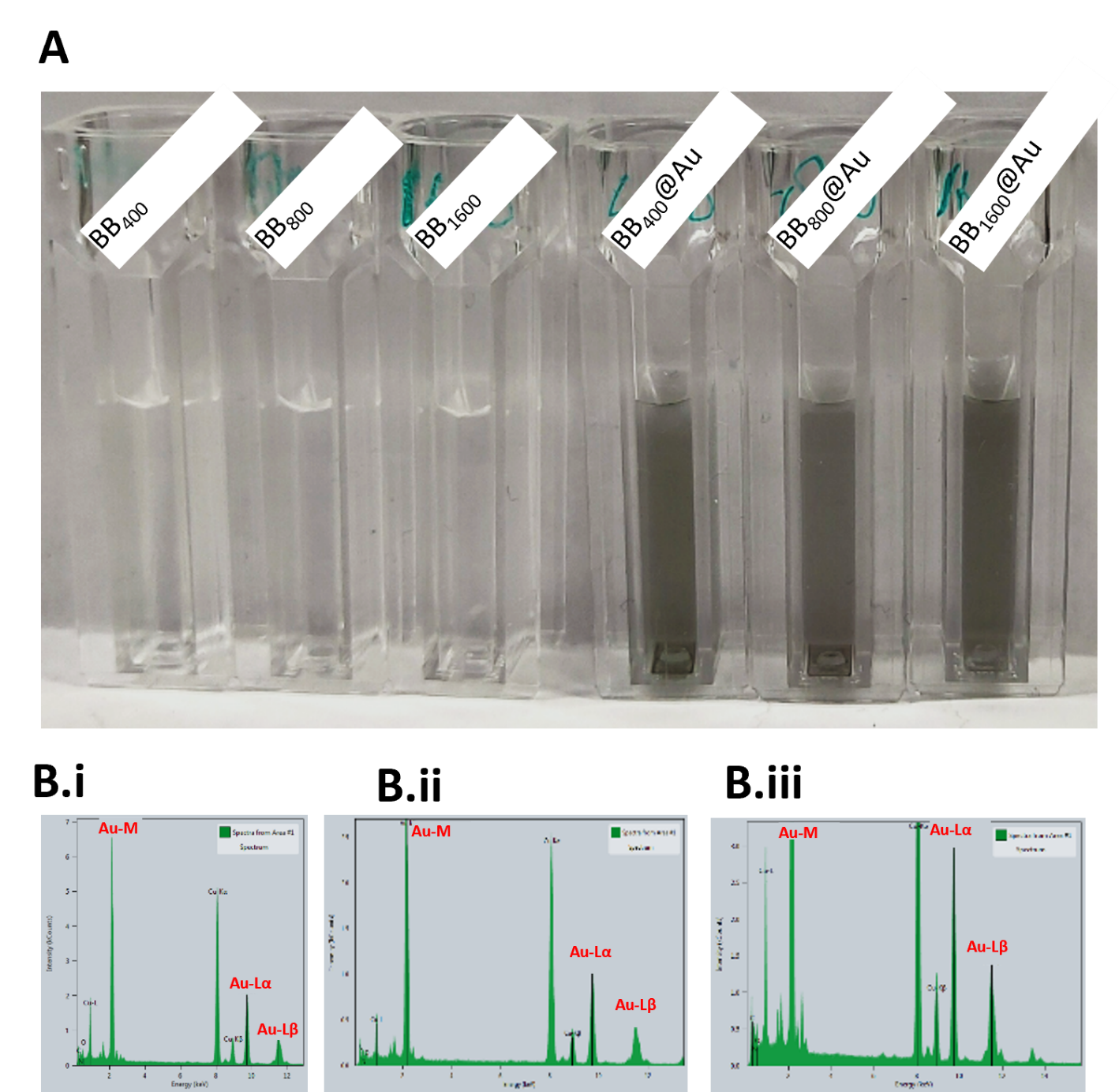
**

**Fig. S7. A)** Digital image showing the BB_400_, BB_800_, BB_1600_, BB_400_@Au , BB_800_@Au and BB_1600_@Au. **B.i, ii, iii)** The quantitative elemental analysis of BB_400_@Au (i), BB_800_@Au (ii)_,_ and BB_1600_@Au (iii).

1. **Evaluation of biocompatibility**

**
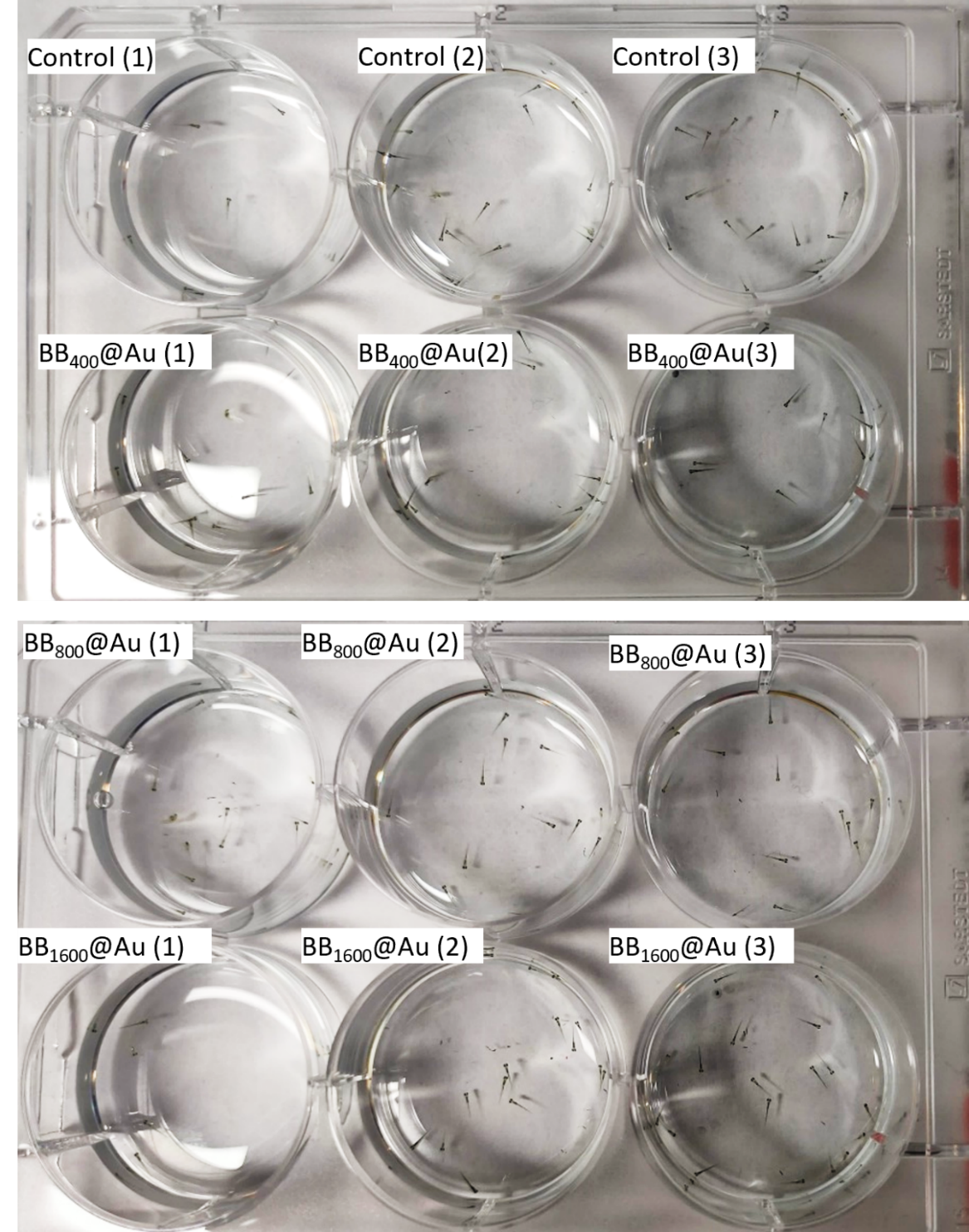
**

**Fig. S8** Digital image showing the zebrafish larvae cultured for 5 days to determine the biocompatibility of BB@Au NPs.

**
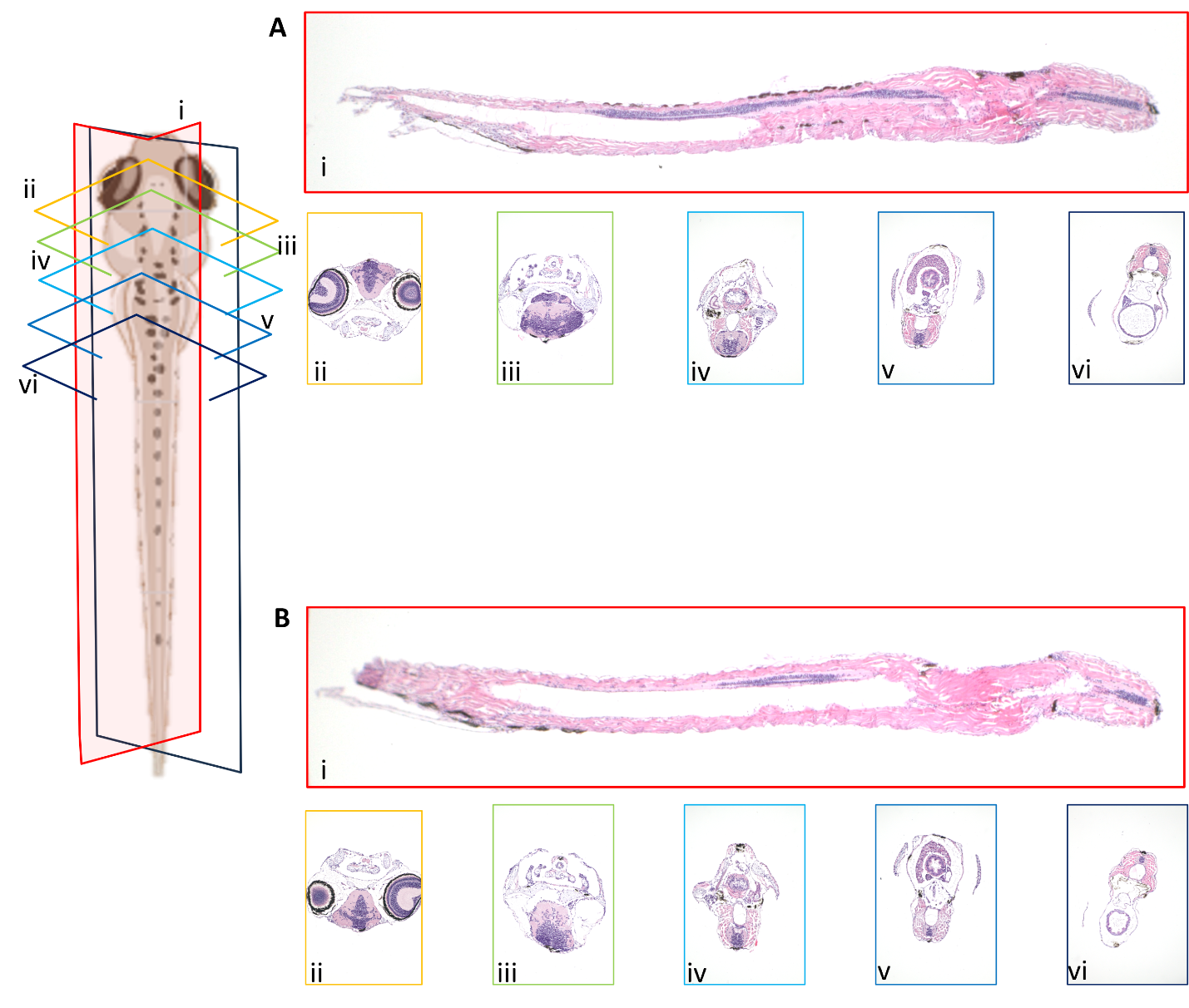
**

**Fig. S9** Transverse and longitudinal sections of zebrafish stained with H&E. **A)** Control, **B)** Zebrafish incubated with BB_400_@Au for 5 days.

**
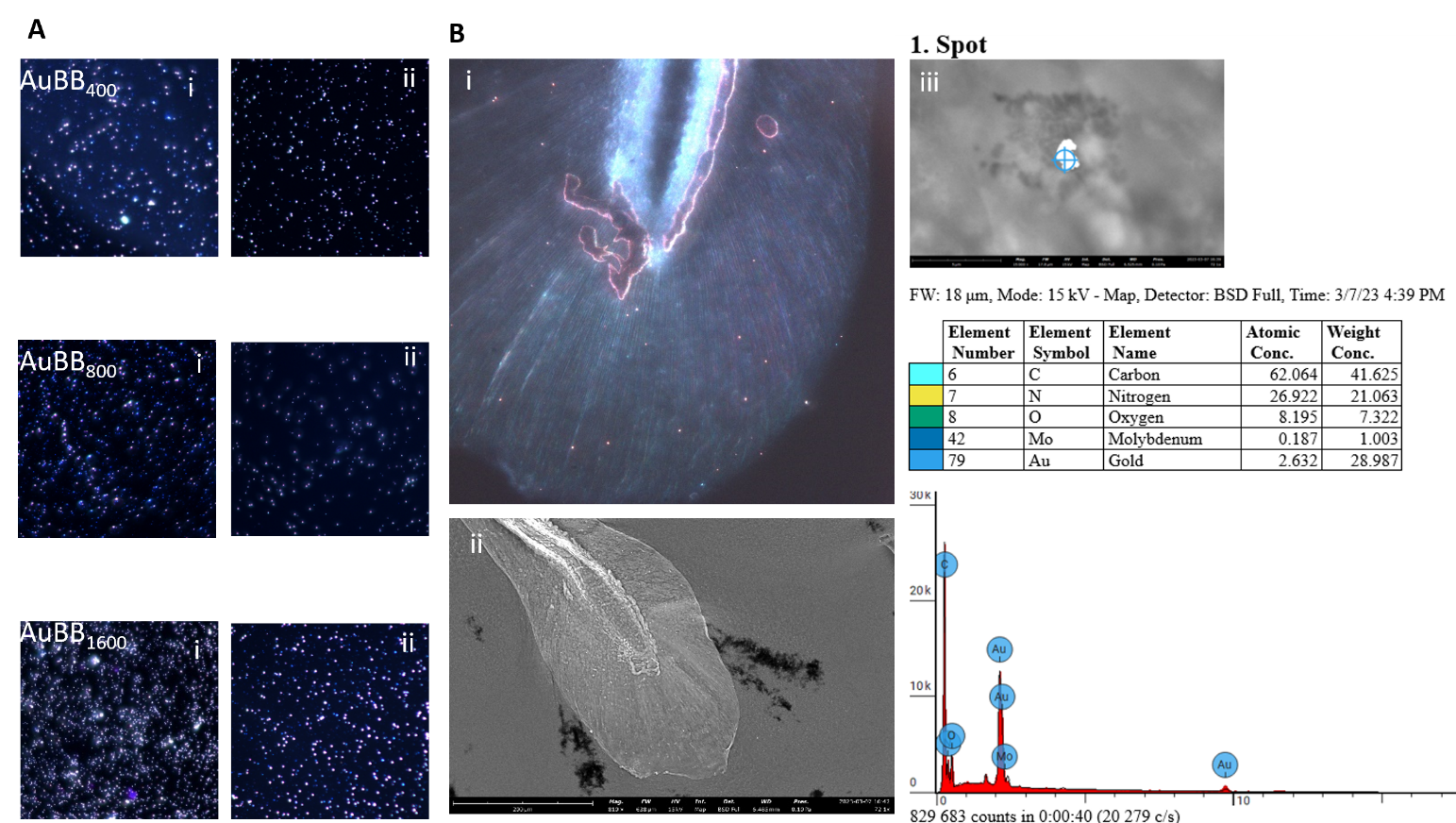
**

**Fig. S10** Side illumination of BB@Au NPs. **A.i,ii)** The side illumination images of BB@Au NPs fixed on glass slide without coverslip (i) and with oil and coverslip (ii). **B.i,ii,iii)** The side illumination image of the whole zebrafish (i) compared with the corresponding SEM image (ii) by confirming the elemental analysis of gold (iii), the bright red spots indicate the BB_400_@Au .

**
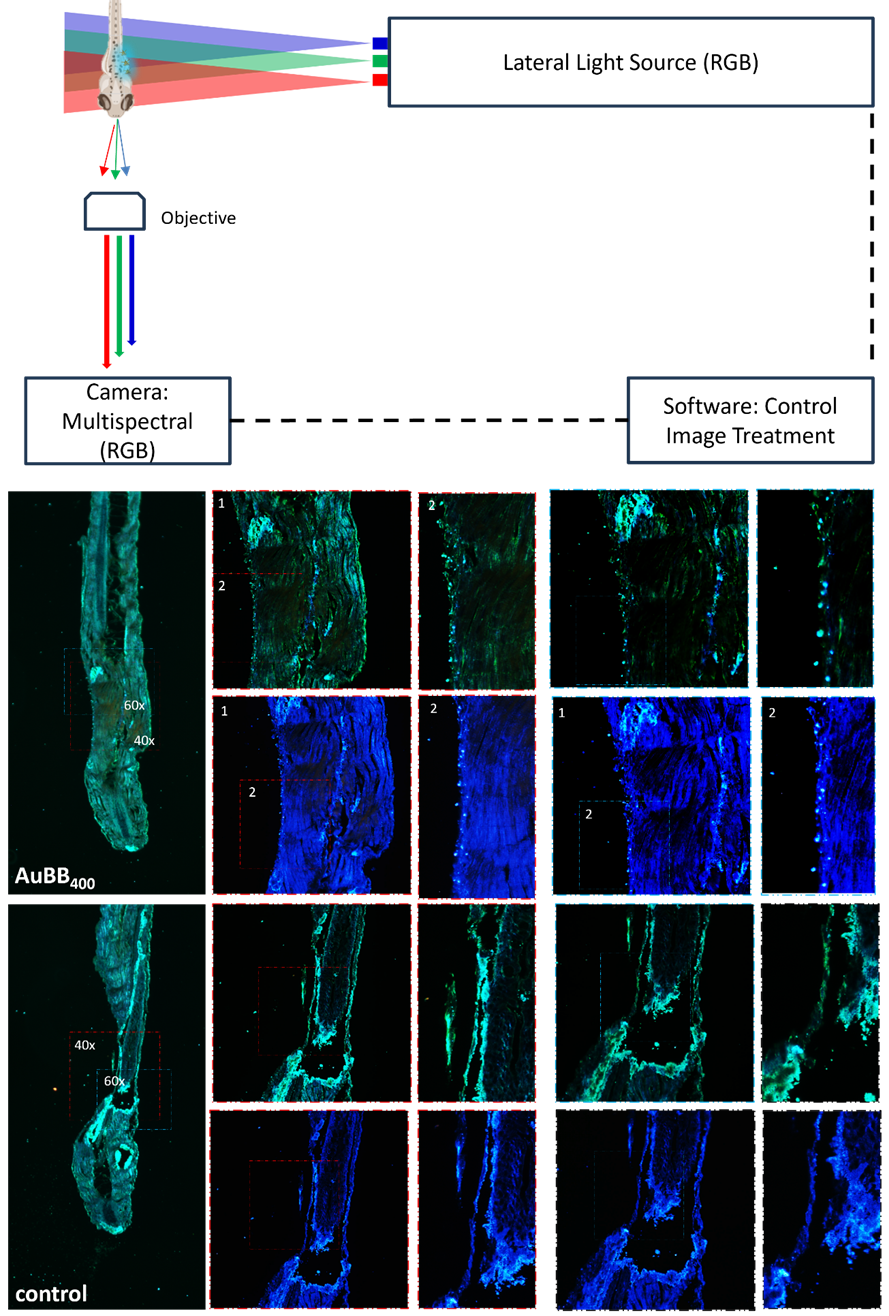
**

**Fig. S11 Schematic showing the process of Side Illumination Microscopy.** It facilitates the visualization of red, green, and blue color scattering plasmonic NPs inside the zebrafish larvae.

1. **Evaluation of photothermal properties**


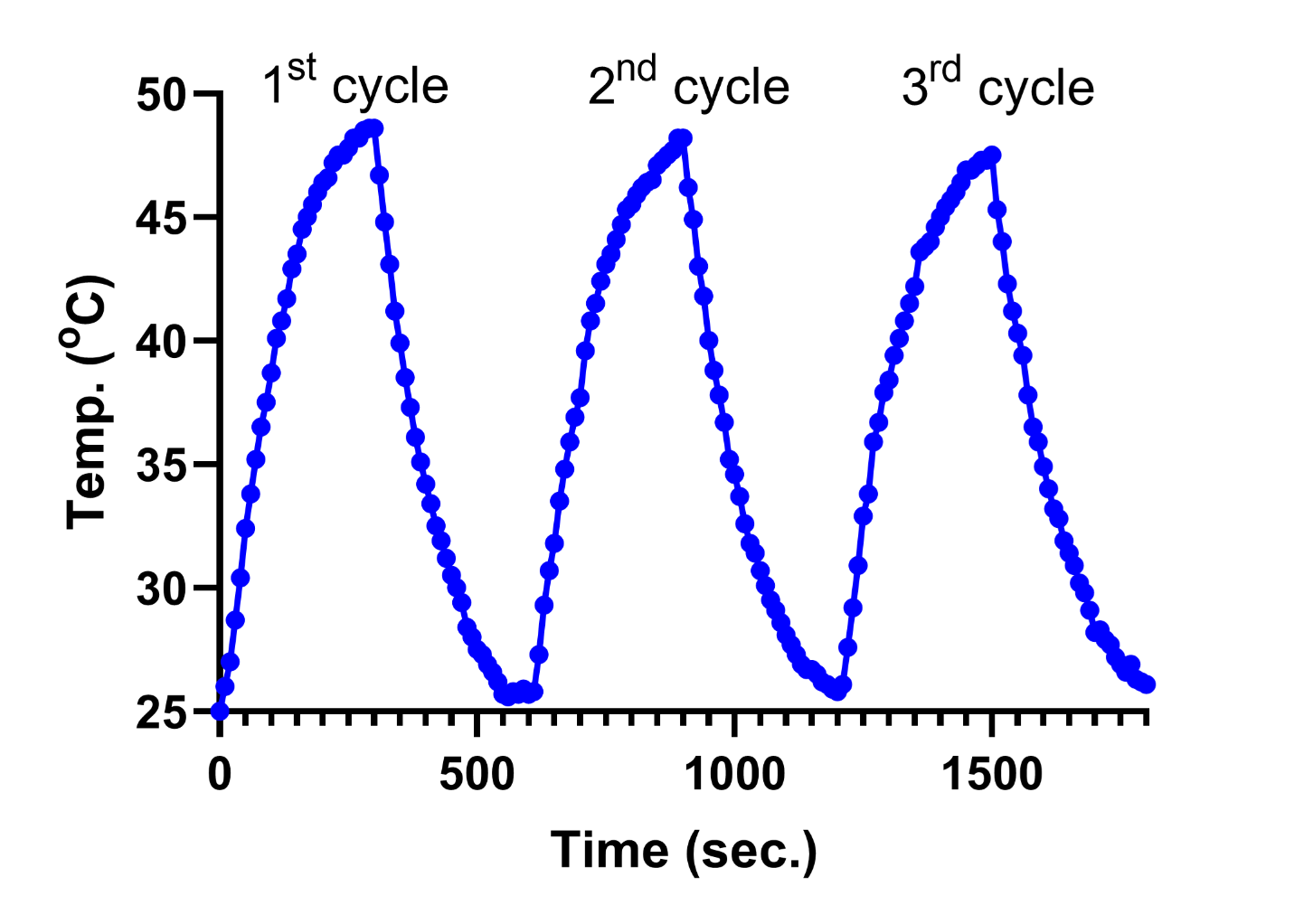


**Fig. S12** The multiple irradiations of BB_400_@Au with NIR-I laser. The maximum temperature achieved during the first cycle was almost maintained in 2^nd^ as well as 3^rd^ cycle.

**
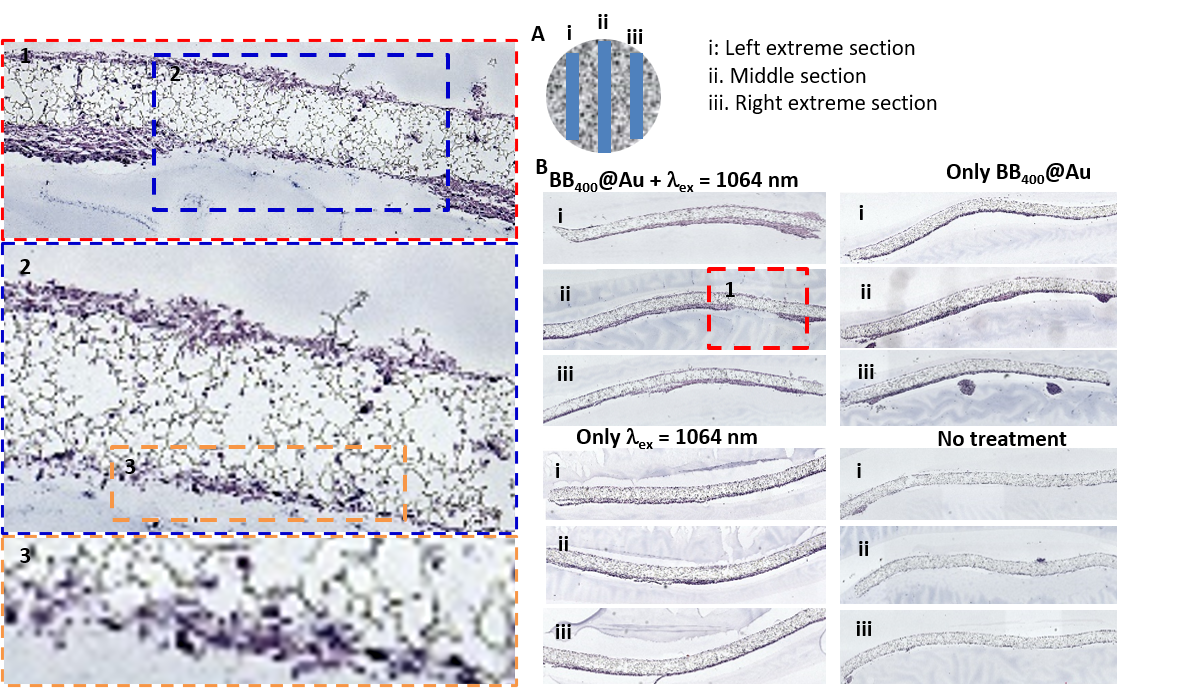
**

**Fig. S13** The longitudinal section of scaffold at the end of the 3D photothermal therapy. **A)** The schematic showing the planned longitudinal sections. **B)** The H&E-stained sections of different groups; the zoomed sections are shown in dashed rectangles.

Fig. S14 Raman spectra recorded at varying concentrations of (MBA, 0–2000 nM) conjugated to BB₈₀₀@Au nanoparticles, highlighting the characteristic MBA peak at 1590 cm⁻¹, red arrow (Left). Calibration curve showing the linear correlation between Raman intensity at 1590 cm⁻¹ and analyte concentration, with linear regression analysis (R² = 0.994) and 95% confidence intervals, dotted lines (Right). The estimated limit of detection was determined to be 12.9 nM, demonstrating the high SERS sensitivity of BB₈₀₀@Au nanoparticles. Data are presented as mean ± SD from replicate measurements (n = 3)

**Photothermal Efficacy calculations:** The photothermal efficiency of BB@Au NPs was calculated following the studies by Roper et al.[1],

$$ƞ=\frac{\mathrm{hS}\left( T_{\mathrm{Max}}-T_{\mathrm{Surr}} \right)-Q_{\mathrm{Dis}}}{I(1-{10}^{-A_{808, 1064}})}$$

Wherein, η is the photothermal efficiency, Q_Dis_ (dissipated heat due to solvent absorption or baseline energy input) was measured independently to be 39.1 mW for NIR-I and NIR-II lasers using water. T_max_-T_surr_ (temperature difference) for BB_400_@Au , BB_800_@Au , BB_1600_@Au was determined to be 14.5 ^o^C, 13.5 ^o^C, 19.5 for NIR-I laser, and 21.3 ^o^C, 19 ^o^C, 20.1 for NIR-II laser, I (power density of the NIR-I and NIR-II lasers) is 500 mW/cm^-2^. A_808,1064_ (intensity of BB@Au NPs' absorbance at 808 and 1064 nm) for BB_400_@Au , BB_800_@Au , BB_1600_@Au was determined to be 0.65, 0.61, 0.78 at 808 nm and 0.85, 0.8, 0.89 at 1064 nm wavelengths, respectively. *hS* was deduced using the following equation[2],

$$hS=\frac{mCp}{\tau s}$$

wherein m (mass of the solution) is 0.3 g and C (heat capacity of the solution) is 4.2 J/g, τ_s_ (time constant) was determined by plotting the time versus negative logarithm of temperature in cooling period. Substituting these values in above equation, *hS* for BB_400_@Au , BB_800_@Au , BB_1600_@Au , was found to be 8.7 mW, 8.6 mW, 7.1 mW for NIR-I laser and 7 mW, 7.2 mW, 7.4 mW for NIR-II laser.

**References**

[1] Roper, D. K.;Ahn, W.; Hoepfner, M. Microscale Heat Transfer Transduced by Surface Plasmon Resonant Gold Nanoparticles. *J Phys Chem C Nanomater Interfaces* **2007**, *111*, 3636-3641.

[2] Roper, D. K.;Ahn, W.; Hoepfner, M. Microscale Heat Transfer Transduced by Surface Plasmon Resonant Gold Nanoparticles. *The Journal of Physical Chemistry C* **2007**, *111*, 3636-3641.
